# Preservation and alteration of inclusion-based calcite-water oxygen isotope and clumped isotope temperature signals in calcite veins

**DOI:** 10.1038/s41598-025-92824-w

**Published:** 2025-03-14

**Authors:** Attila Demény, László Rinyu, Yuri Dublyansky, Bernadett Bajnóczi

**Affiliations:** 1 Institute for Geological and Geochemical Research, HUN-REN Research Centre for Astronomy and Earth Sciences, Budaörsi Út 45, Budapest, 1112 Hungary; 2 CSFK, MTA Centre of Excellence, Konkoly Thege Miklós Út 15-17, Budapest, 1121 Hungary; 3 Isotope Climatology and Environmental Research Centre, HUN-REN Institute for Nuclear Research, Bem Tér 18/C, Debrecen, 4026 Hungary; 4 Institute of Geology, University of Innsbruck, Innrain 52, 6020 Innsbruck, Austria

**Keywords:** Hydrothermal calcite, Oxygen isotope thermometry, Fluid inclusion water, Calcite, Clumped isotope thermometry, Red calcite veins, Hungary, Solid Earth sciences, Geochemistry

## Abstract

Knowledge of the formation temperatures of geological deposits is essential for investigating their genesis. Oxygen isotope thermometry (OIT), using the temperature dependence of oxygen isotope fractionation between host carbonate mineral and mineral-forming water trapped in fluid inclusions, and clumped isotope thermometry, based on the degree of ^13^C and ^18^O clumping, are receiving increasing interest. However, only a few studies have applied combinations of these methods, and their databases are limited. In this study, we compare OIT and clumped isotope temperatures obtained for 18 samples from Mesozoic to early Cenozoic calcite veins. Our analysis indicates that the formation temperatures were preserved in the clumped isotopic compositions (16–45 °C), whereas the OIT temperatures were shifted to lower temperatures (− 2 to 33 °C). An OIT temperature shift occurred, due to a retrograde oxygen isotope exchange between the fluid inclusion water and the host calcite. These results imply that the retrograde isotope exchange should be taken into consideration, even for low-temperature carbonate deposits, if a sufficiently long time is available.

## Introduction

Two commonly used methods for determining the formation temperature of natural calcite are calcite-water oxygen isotope thermometry (hereafter OIT) and clumped isotope thermometry (hereafter ∆_47_-thermometry). The former method is based on the temperature dependence of oxygen isotope fractionation between calcite and the parent water, which has been determined by theoretical calculations e.g.,^1^ empirical observations of natural calcite deposits with known formation temperatures^2–6^, and laboratory experiments^7–10^. The general equation for the temperature dependence of calcite-water oxygen isotope fractionation is 1000·lnα = A/T + B for low temperatures (below approximately 100 °C) and 1000·lnα = A/T^2^ + B for higher temperatures. The fractionation factor α is the ratio of the ^18^O/^16^O ratios in calcite (cc) and water (w), which can also be expressed as δ^18^O values, normalized to the same standard (in this case, V-SMOW): α = (1000 + δ^18^O_cc_)/(1000 + δ^18^O_w_). The A and B constants of the general equations vary significantly in different calibrations mentioned above. With an appropriate calibration selected (e.g., speleothem-based calibration^4^ for a speleothem study), the temperature can be calculated if the δ^18^O values of both the calcite and the mineral-forming water are known. This means that, in addition to the oxygen isotope composition of calcite, which can be readily measured, one needs to know the composition of the mineral-forming water. The latter can be either estimated (e.g., Shackleton and Kenneth^11^ for global ocean water composition and marine carbonates) or measured in fluid inclusion-hosted water^12^. A significant uncertainty in the application of the OIT method may be associated with the equation’s “fluid inclusion water” term. In all currently existing analytical methods, fluid inclusion water for stable isotope analysis is obtained by the crushing of aliquots of calcite (hundreds of mg to several g). The released water represents a mix of the contents of all inclusions in the crushed calcite (commonly quantitatively biased towards the content of the largest inclusions). Stable isotope measurements obtained from such a mix can be used for OIT calculations if the crushed sample contains only primary inclusions (or, more realistically, when primary inclusions overwhelmingly dominate the mix). In this case, the measured δ^18^O values faithfully reflect the isotopic composition of the mineral-forming water. The prevalence of primary fluid inclusions must be ascertained by the careful study of fluid inclusion petrography. Further uncertainty in OIT arises from the fact that the stable isotope composition of inclusion-hosted water may have been altered by diagenetic processes^13^.

Another method that uses fluid inclusions and is not affected by the diagenetic alteration of calcite is nucleation-assisted fluid inclusion microthermometry. This method typically yields more precise and accurate temperatures than the isotope-based methods^14^. However, this method is not applied in this study, so it will not be discussed in greater detail.

Clumped isotope thermometry does not require knowledge of water composition. This method takes advantage of the preferential bonding (“clumping”) of heavy carbon (^13^C) and oxygen (^18^O) isotopes in the CaCO_3_ structure beyond the stochastic equilibrium. The heavy isotope clumping (^13^C^18^O^16^O in CO_2_, which has a molecular mass of 47) is expressed by the Δ_47_ value^15^, which represents the difference between the random ^13^C^18^O distribution and the measured composition, and is temperature-dependent^15^. Similar to OIT, several calibrations exist for the temperature dependence of Δ_47_ (e.g., based on laboratory experiments^10^ or on observations of natural formations^16^). Various equations have been compiled by a recent calibration^17^, which will be used in this study. Clumped isotope thermometry also has a significant source of uncertainty, specifically, the possible effect of kinetic fractionation that is primarily observed in carbonates, whose formation is associated with fast degassing or absorption of CO_2_^18^. This is why carbonates that deposit from water films, such as subaerial speleothems, seldom yield accurate clumped isotope temperatures, whereas carbonates that form subaqueously, where CO_2_ is degassed slowly (e.g., hydrothermal veins, spars, and subaqueous speleothems^12,14^), may provide accurate ∆_47_ temperature data.

Both of the isotope-based thermometry methods described above (OIT and ∆_47_-thermometry) may be affected by a retrograde isotope exchange during the cooling of high-temperature (> 100 °C) carbonate formations^19,20^. This factor is another potential source of uncertainty. As a result, the possibility of the retrograde exchange must be considered during the evaluation of temperatures obtained by OIT and ∆_47_-thermometry.

**Fig. 1:**
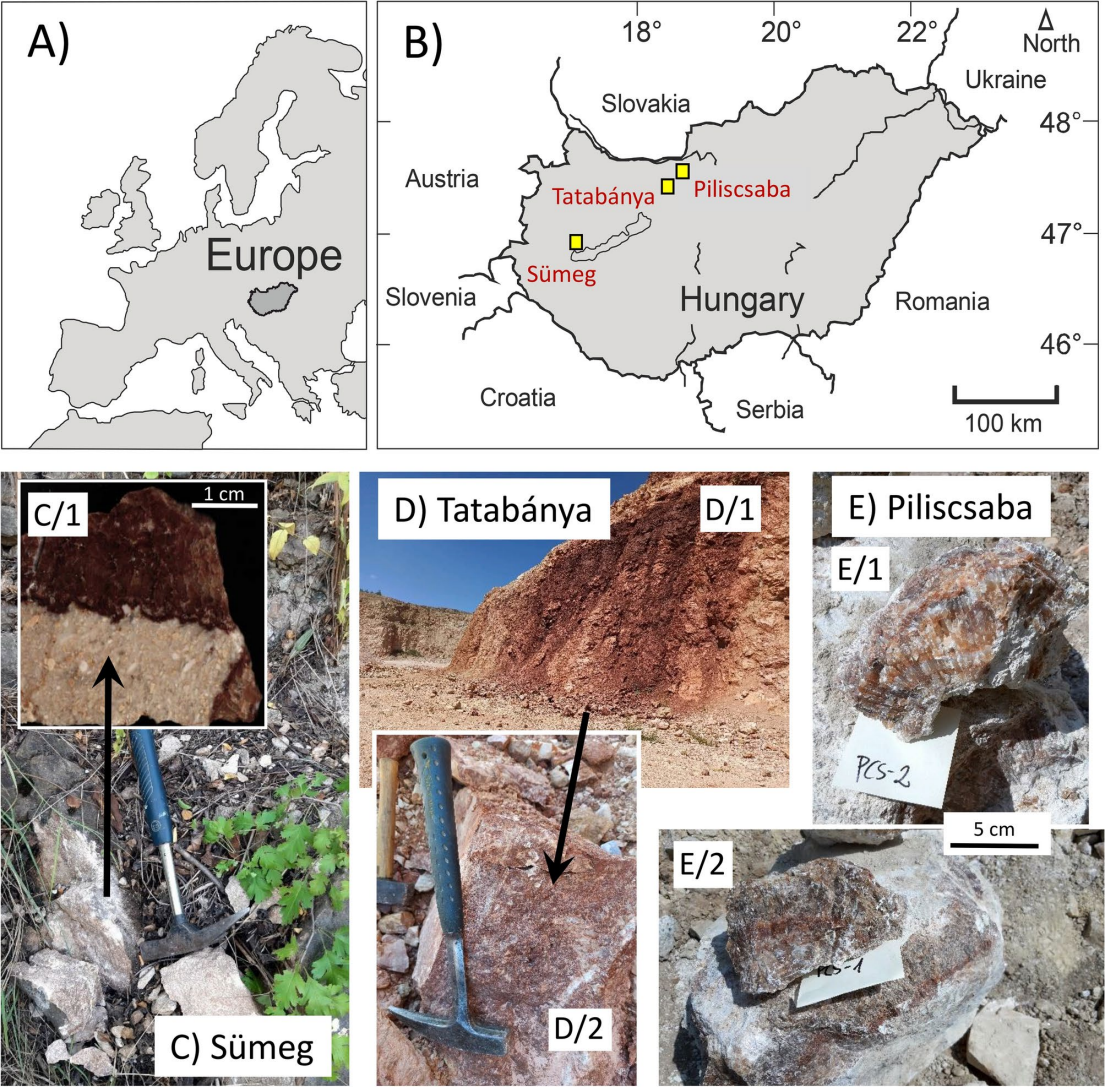
(**A**) and (**B**) The studied red calcite locations. (**C**) Red calcite vein of the Sintérlap quarry, Sümeg. The insert (C/1) shows a polished section of the limestone-vein contact. (**D**) The wall of the Keselő Hill quarry at Tatabánya (D/1), where the sampled red calcite block (D/2) was collected. (**E**) Red calcite block sampled in the Piliscsaba-Jászfalu quarry. E1: sample PCS-2; E/2: sample PCS-1.

In this study, we applied the OIT and ∆_47_-thermometry techniques to red calcite veins in Hungary. The veins were formed after the Late Triassic and before the Oligocene due to an influx of fluids bearing Fe oxyhydroxide minerals from bauxitic deposits into fractures^21^. The origin of red calcites is controversial. An early study determined their formation temperature to be ca. 150 °C^22^, but a later study, based upon fluid inclusion petrography, inferred a significantly lower formation temperature (< 50 °C)^21^. In our study, we collected samples of red calcite veins from three locations (Fig. 1), conducted detailed calcite and fluid inclusion petrographic investigations, and performed conventional and clumped stable isotope analyses of calcites and inclusion-hosted waters. In addition to the determination of formation temperatures, we also evaluated the long-term preservation of temperature signals associated with the two thermometric techniques.

## Results

### Calcite fabrics and fluid inclusion petrography

Detailed descriptions of calcite and fluid inclusion petrographic analyses are provided in the Supplementary Material, whereas in this section, the main features are summarized. The red calcite veins of the Sümeg and Piliscsaba localities are composed of elongated, columnar crystals bounded by compromise growth boundaries (compact columnar fabric^23^). The red calcite at the limestone-vein contact at the Sümeg location (sample SÜ-4) has variable fabrics, ranging from columnar to mosaic. The dark red calcite of the Tatabánya occurrence is either characterized by isometric euhedral to subhedral crystals that are densely packed into a mosaic fabric (sample VK-1) or has a complex build-up featuring euhedral crystals and columnar overgrowth. The growth zones are pronounced in individual crystals. The pale red sparry calcite of Tatabánya (sample VK-2) has a compact columnar fabric. Almost all of the studied red calcite samples show signs of mechanical stress in the form of lamellar twinning, particularly in the latest generations of calcite crystals, which is consistent with the tectonic reopening of fissures^24^. The samples where mechanical stress is most evident and twinning is most significant are PCS-1, SÜ-3, SÜ-4, VK-1, and VK-2.

**Fig. 2:**
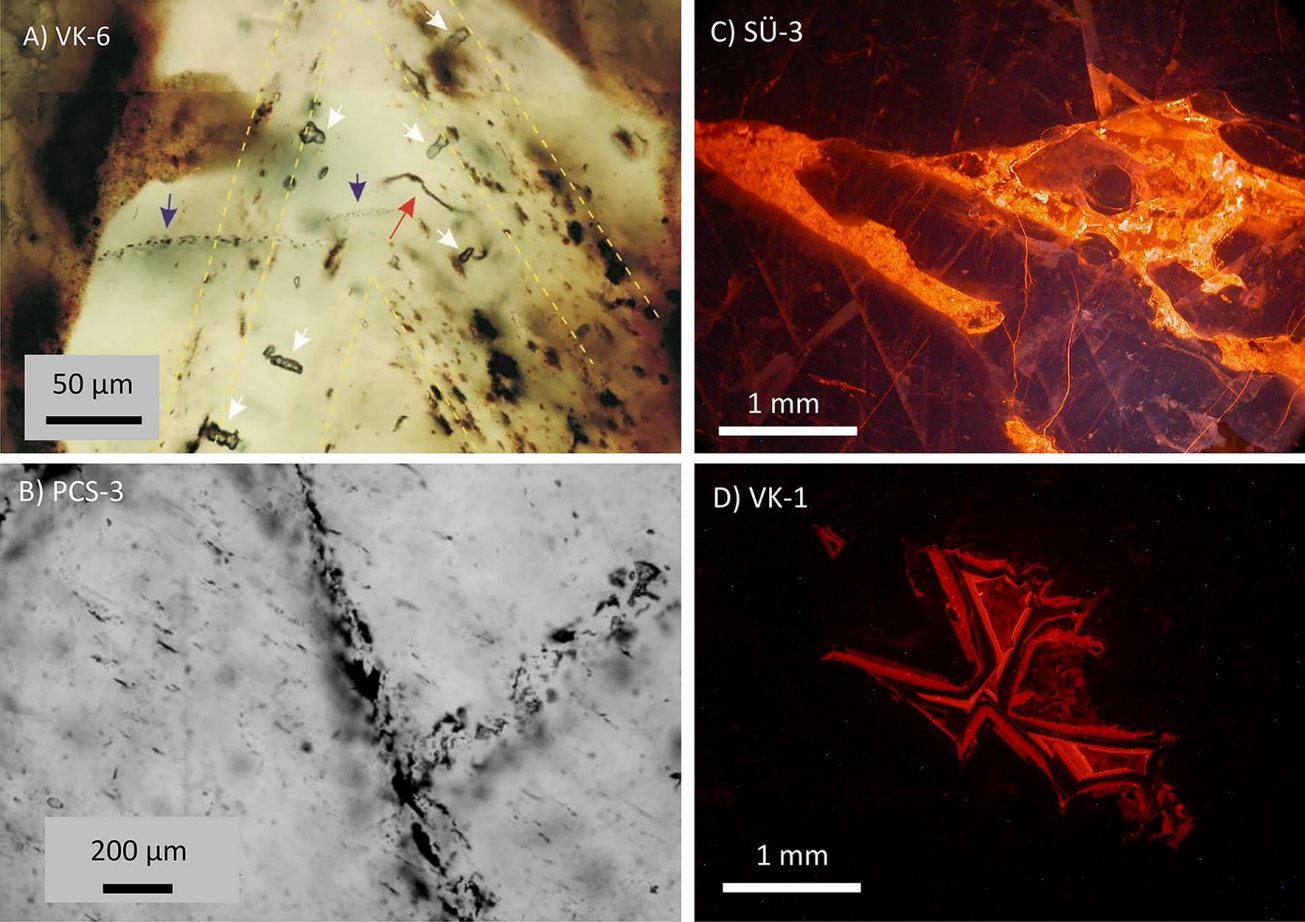
Fluid inclusions and cathodoluminescence of selected samples. *(***A**) Distribution and character of fluid inclusions in sample VK-6 from the Tatabánya Keselő-hegy quarry. Numerous and volumetrically dominant primary inclusions (white arrows) are aligned along calcite crystal growth zones (emphasized by yellow dashed lines) and/or have pronounced ”directional” waisted morphology. Secondary inclusions (blue arrows) form curvilinear trails; they are volumetrically subordinate. The filament (a remnant of filamentous fungi) is shown by a red arrow. (**B**) Several small secondary inclusions trapped along cleavage fractures in sample PCS-3 (Piliscsaba-Jászfalu quarry). (**C**) Cathodoluminescence image of sample SÜ-3, taken from the boundary between subsamples SÜ-3 sparry and SÜ-3 dark. (**D**) Cathodoluminescence image of sample VK-1, showing a pocket of luminescing zoned diagenetic calcite.

In most of the examined samples, primary single-phase aqueous inclusions were the dominant type by volume, although small numbers of secondary inclusions were also present. Inclusion trails along cleavage fractures, identified as secondary, were most prominent in samples PCS-2 and PCS-3. In contrast, secondary inclusions in samples VK-1 and VK-6 were volumetrically minor (Supplementary Material). In all the samples analyzed in this study, primary fluid inclusions were shown to be volumetrically dominant (Fig. 2A), although some parts of samples contained secondary inclusions (Fig. 2B). The stable isotope properties of the waters released by the bulk crushing of samples can therefore be attributed to the waters from which the calcite formed. Based on the character of primary fluid inclusions (single-phase aqueous), the formation temperatures of red calcites are unlikely to exceed approximately 50 °C. The curved tubular features observed in several samples (e.g., Fig. 2A) can be interpreted as remnants of organic filaments^25^. They support the notion of a relatively low formation temperatures and the involvement of surface-derived solutions.

Cathodoluminescence images were taken from polished samples (see Supplementary Material for the complete set of pictures). Luminescent diagenetic calcite is most prominently observed in sample SÜ-3 within a fracture zone (Fig. 2C) separating the sparry and dark red subsamples. In these subsamples, smaller amounts of luminescent calcite are present, primarily along grain boundaries. In the other samples, luminescent calcite is typically restricted to grain boundaries and isolated pockets (see Fig. 2D and the Supplementary Material) and is volumetrically minor.

### Stable isotopic compositions of calcites and fluid inclusion waters

Table 1 lists the water contents and stable carbon and oxygen isotopic compositions of red calcites, as well as the stable hydrogen and oxygen isotopic compositions of inclusion-hosted waters. The calcite veins from the three locations differ significantly in the volume of fluid inclusions (expressed as the H_2_O content in calcite), calcite δ^13^C and δ^18^O values, isotopic composition of fluid inclusion water (δ^2^H_fi_ and δ^18^O_fi_), and clumped isotope temperatures. The Sümeg samples exhibited higher water contents (741 to 3270 ppm) and higher δ^2^H_fi_ and δ^18^O_fi_ values (–29 ± 7 ‰ and –4.2 ± 0.6 ‰, respectively) compared to the Tatabánya and Piliscsaba samples (H_2_O from 654 to 2312 ppm, δ^2^H_fi_ from –63 to –45 ‰, and δ^18^O_fi_ from –9.8 to –3.7 ‰). Another notable difference is found in the δ^13^C values of the calcites, with the Sümeg vein showing a slight enrichment in ^13^C (–4.8 ± 0.9 ‰) compared to the other two locations (–6.5 ± 0.5 ‰ at Tatabánya and –10.2 ± 1.2 ‰ at Piliscsaba). However, it is important to note that the previously published δ^13^C data^21,22^ have a slightly lower average (–7.8 ± 1.4 ‰) for the Tatabánya location. Nevertheless, the δ^13^C and δ^18^O values of the calcites in this study are consistent with the previous results^21,22^, indicating that despite the different veins and collection periods, the isotopic compositions obtained here are representative of the locations.

**Table 1:** Stable isotope compositions (in ‰) of calcite (“cc”) and fluid inclusion water (“fi”), as well as calcite-water oxygen isotope temperatures (T(OIT), in °C), calculated using the equation of Johnston et al^5^.

	H_2_O ppm	δ^2^H_fi_	δ^18^O_fi_	δ^13^C	δ^18^O_cc_	T(OIT)	±	T(Δ_47_)	95%CI
*Sümeg*									
SÜ-1a	3270	−23.4	−4.4	−5.3	25.7	21.5	5.0	37.6	14.7
SÜ-2	3061	−22.1	−3.4	−4.6	25.5	27.5	5.2	17.4	12.7
SÜ-3 sparry	741	−32.2	−5.1	−3.8	25.9	17.1	4.9	29.2	16.1
SÜ-3 dark	1369	−39.0	−4.3	−4.4	26.0	20.4	5.0	15.6	14.5
SÜ-4	1974	−27.3	−4.0	−6.1	25.9	22.7	5.1	31.1	16.6
*Tatabánya*									
VK-1	1239	−45.3	−5.0	−5.9	26.3	15.7	4.8	29.8	9.7
VK-2	741	−47.8	−6.3	−7.0	26.2	10.3	4.6	29.8	10.6
VK-4	751	−63.0	−9.2	−6.1	26.0	−1.5	4.3	45.2	9.8
VK-6	2312	−56.0	−5.9	−6.9	26.2	12.1	4.7	37.1	9.0
*Piliscsaba*									
PCS-1–1	1861	−59.7	−8.3	−10.1	24.0	10.7	5.0		
PCS-1–2	1438	−57.6	−7.3	−9.2	24.2	14.2	4.8	29.2	9.3
PCS-1–3	1051	−56.7	−9.8	−8.8	24.1	3.7	4.4	32.5	9.3
PCS-1–4	2063	−47.4	−6.5	−11.5	24.4	17.4	4.9	27.5	9.0
PCS-1–5	2091	−49.4	−5.2	−10.3	24.0	25.9	5.2	26.2	8.9
PCS-2–1	2060	−60.2	−9.1	−7.7	24.1	6.7	4.5	30.1	7.0
PCS-2–2	2266	−49.1	−3.7	−11.3	24.0	33.4	5.4	22.7	6.8
PCS-2–3	1515	−53.5	−7.1	−11.3	24.4	14.6	4.8	34.8	7.4
PCS-3–1	1993	−47.4	−5.5	−10.6	24.7	20.5	5.0	35.1	7.4
PCS-3–2	654	−46.9	−8.1	−10.8	24.7	8.6	4.6	24.8	6.8

The ± values are propagated errors (see text). Also included are the clumped isotope temperatures (T(Δ_47_), in °C), calculated using the D47crunch algorithm^38^. δ^2^H_fi_, δ^18^O_fi_ and δ^18^O_cc_ values are relative to V-SMOW, and δ^13^C values are relative to V-PDB.

### Comparison of the OIT and Δ_47_ thermometry results

**Fig. 3:**
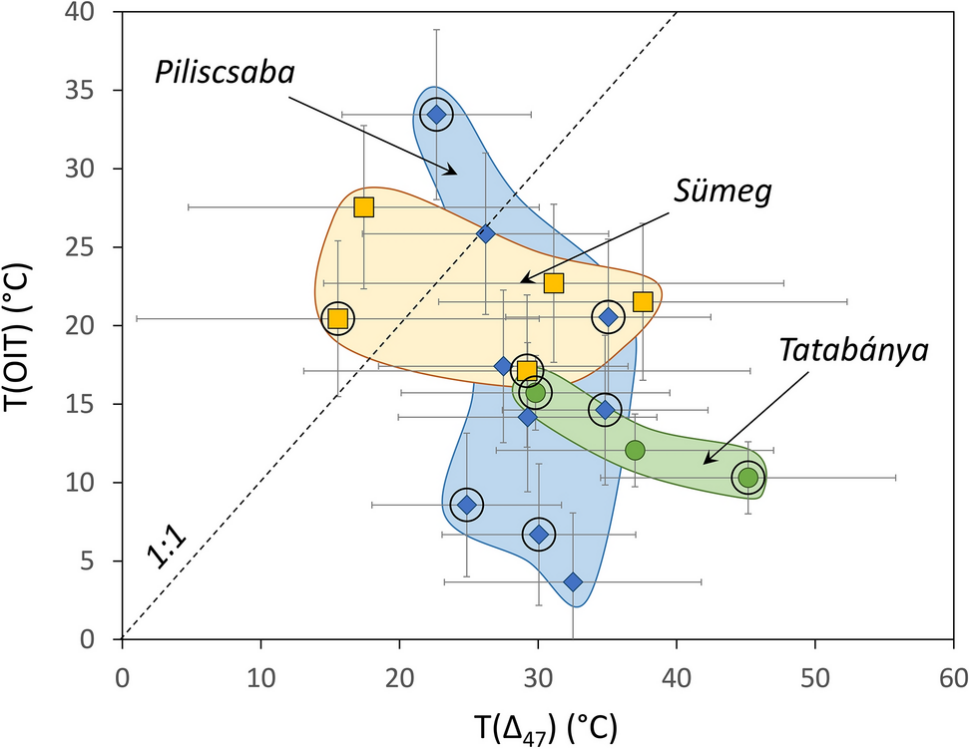
Comparison of temperatures obtained for red calcites by clumped isotope (Δ_47_) and oxygen isotope thermometry (OIT) methods. Uncertainties are worst-case scatters using analytical errors for T(OIT) values and 95% confidence interval values for T(Δ_47_) data. Empty circles: samples with the highest amounts of cathodoluminescent calcite and secondary inclusions.

The ∆_47_ temperatures—denoted T(Δ_47_) in the followings—were obtained using the calibration equation of Anderson et al.^17^. The Sümeg samples yielded slightly lower T(Δ_47_) values (26.2 ± 9.4 °C) compared to the Tatabánya and Piliscsaba samples (35.5 ± 7.3 °C and 29.2 ± 4.3 °C, respectively). The OIT temperatures—denoted T(OIT) in the followings—were calculated using measured δ^18^O values of calcite and inclusion-hosted water. Calculations returned much lower, compared to the T(Δ_47_) values, with averages of 21.9 ± 3.8 °C, 9.2 ± 7.4 °C, and 15.6 ± 9.1 °C for the Sümeg, Tatabánya, and Piliscsaba occurrences, respectively. The T(Δ_47_) and OIT results are compared in Fig. 3.

## Discussion

As Table 1 and Fig. 3 show, both the T(Δ_47_) and the T(OIT) values suggest formation temperatures below 50 °C, aligning with the petrographic observations on the fluid inclusions. These data support the interpretations of Győri et al.^21^, and therefore, a magmatic hydrothermal origin for the red calcites^22^ should be rejected. The notable difference between the T(Δ_47_) and the T(OIT) values warrant further investigation. The data distribution in Fig. 3 may be explained (1) by increasing the T(Δ_47_) values due to post-formational heating, or (2) by different degrees of alteration and related decreases of the T(Δ_47_) and the T(OIT) values, or (3) by decreasing the T(OIT) values by post-formational oxygen isotope exchange between the inclusion-hosted water and the host calcite. The shift of the T(Δ_47_) values to higher temperatures would require post-formational heating, which would also result in changes in the OIT temperatures due to calcite-water oxygen isotope exchange. No petrographic evidence of such heating was detected in the samples analyzed in this study.

**Fig. 4:**
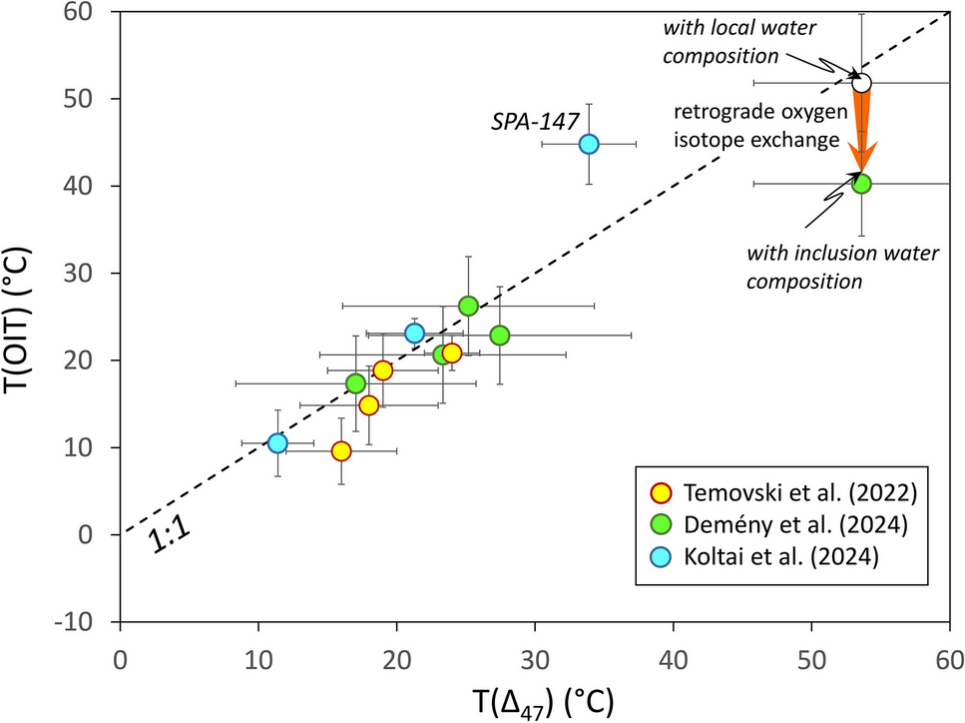
Comparison of temperatures obtained by clumped isotope (Δ_47_) and oxygen isotope thermometry (OIT) methods for calcite coatings on marbles^27^, subaqueous calcite deposits^12^, and sparry calcite^14^ formed in caves. Uncertainties are the same as in Fig. 3 of this study and for the Temovski et al.^27^ data, and 1SD (OIT) and 1SE (T(Δ_47_)) values for literature data^12,27^. The high T(OIT) value of sample SPA-147 was interpreted as due to δ^18^O_cc_ inhomogeneity^14^ and hence it can be considered to be an outlier.

**Fig. 5:**
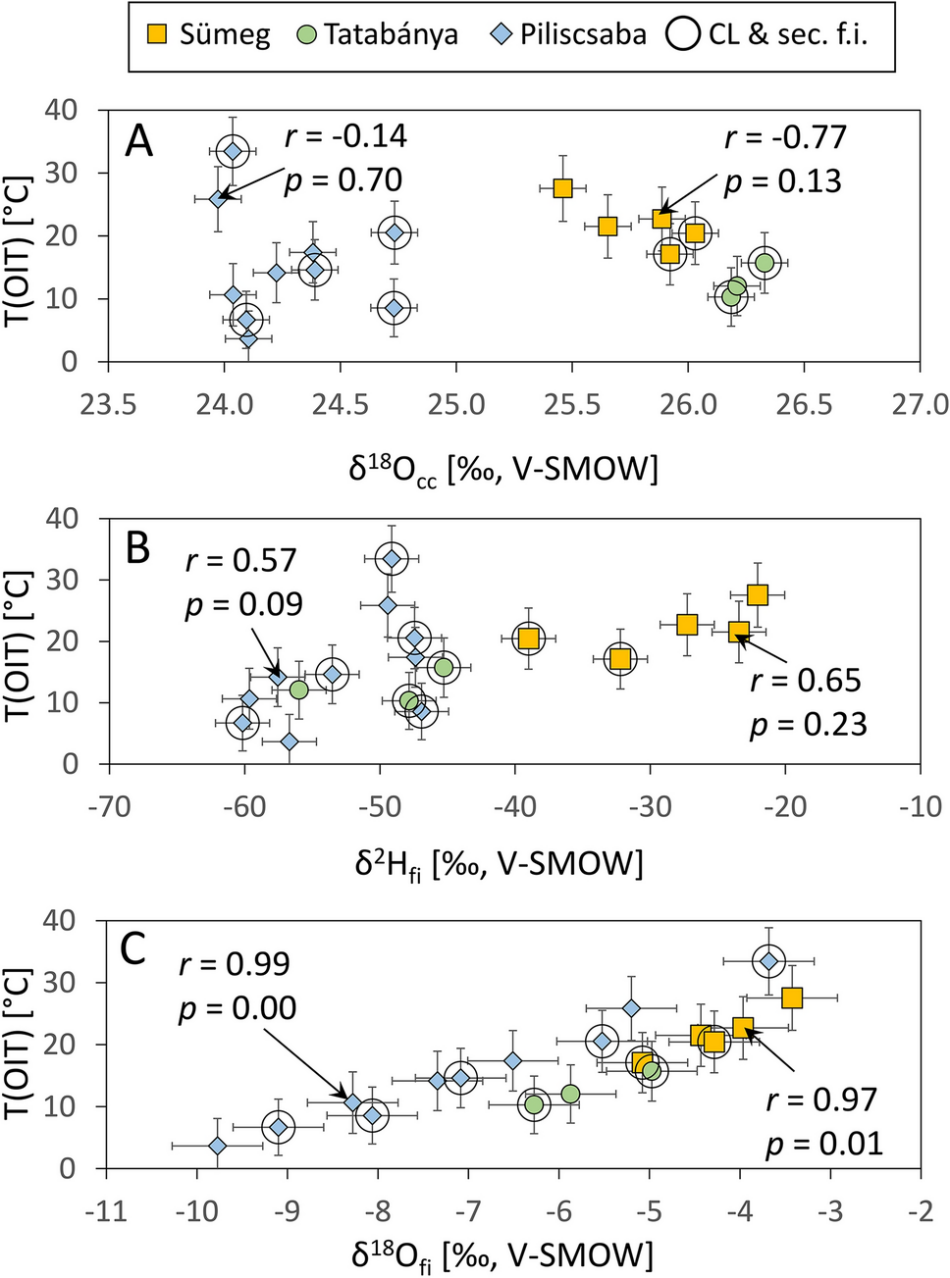
Calcite-water oxygen isotope fractionation temperatures—T(OIT)—compared with (**A**) oxygen isotopic compositions of calcites (δ^18^O_cc_), and (**B**) stable hydrogen and (**C**) oxygen isotope compositions of inclusion-hosted waters (δ^2^H_fi_ and δ^18^O_fi_, respectively). “CL & sec. f.i.”: samples with the highest amounts of cathodoluminescent calcite and secondary inclusions. Uncertainties are 0.1 ‰ for δ^18^O_cc_, 0.5 ‰ for δ^18^O_fi_, errors were calculated using error propagation for the T(OIT) values. Linear correlations are shown for the Sümeg and the Piliscsaba samples. The number of data for the Tatabánya location (n = 3) did not allow for statistical analysis.

The shift of the OIT temperatures toward lower values caused by oxygen isotope exchange between calcite and fluid inclusion water would not be expected in our samples, as their low formation temperatures render calcite-water oxygen isotope exchange negligible^26^. As previous studies^12,13,27^ have shown, relatively low-temperature calcite formations appear to preserve the original T(OIT) and T(Δ_47_) values (Fig. 4). However, an example of a retrograde negative T(OIT) shift of ca. 10 °C was reported by Demény et al.^12^ for subaqueous calcite that precipitated at ca. 50 °C and subsequently resided at 23 °C in a cave lake (Fig. 4). Nooitgedacht et al.^19^ have also concluded that, unless significant water–rock exchange occurs, clumped isotope compositions are more resistant to alteration during cooling compared to the calcite-water oxygen isotope system. The reasoning above suggests that the observed shift in T(OIT) values toward lower temperatures, as shown in Fig. 3, is linked to post-depositional stable isotope exchange, which affects the T(OIT) system. The T(Δ_47_) values can be considered as representative of the primary formation temperatures of the studied red calcites, ranging from 16 to 45 °C. One sample (VK-4 from the Tatabánya location) was excluded as it yielded unrealistically low T(OIT) = –1.5 °C. The T(OIT) shows no correlation with the δ^18^O_cc_ values of the host calcite (Fig. 5A). In contrast, it shows positive correlation with both δ^2^H_fi_ and δ^18^O_fi_ (Fig. 5B,C).

Although a general positive correlation between T(OIT) and δ^2^H_fi_ is observed in the composite dataset, the correlation must be assessed separately for samples from different locations, as their ages may differ^21^, and thus their commonality cannot be assumed. The correlations were found to be non-significant (*p* > 0.05) for the Sümeg and the Piliscsaba datasets (Fig. 5B). Samples with signs of mechanical stress and/or containing secondary fluid inclusion (open circles in Fig. 5B) do not show systematic difference from other samples.

T(OIT) values show good positive correlations with the δ^18^O_fi_ values (Fig. 5C). The T(OIT) values are calculated from measured δ^18^O_cc_ and the δ^18^O_fi_. The lack of correlation T(OIT)-δ^18^O_cc_, and strong correlation T(OIT)-δ^18^O_fi_, indicate that that the decrease of OIT temperatures relative to the T(Δ_47_) is caused by the negative alteration of δ^18^O_fi_.

The bulk oxygen isotope composition of the fluid inclusion-hosted water can be altered by a number of processes, such as: (1) partial loss of fluid inclusion water by diffusion; (2) fluid inclusion refilling—complete or partial replacement of the original mineral-forming water with post-formational fluids; (3) entrapment of secondary inclusions; and (4) post-formational oxygen isotope exchange between the host calcite and the fluid inclusion water.

Signs of mechanical stress have been detected in some of the samples, which may suggest some release of inclusion water. Such release, however, is not expected to result in alteration of isotope compositions of preserved inclusions (only the bulk water content would be expected to decrease). A partial loss of fluid inclusion water by diffusion, would cause preferential removal of the light isotope ^16^O (and, respectively, shift of δ^18^O_fi_ values toward more positive values). Additionally, diffusive loss of water would affect both the δ^2^H_fi_ and δ^18^O_fi_ values, which would show the negative correlation with the water contents. This has not observed in the present dataset. Accordingly, the diffusive loss of water can be ruled out as a potential cause of the negative δ^18^O_fi_ shifts.

Post-formational solutions can refill the pre-existing primary inclusions or trapped in secondary fluid inclusions. If stable isotope compositions of these solutions differ significantly from those of the mineral forming waters originally trapped in primary inclusions, the bulk isotopic composition of water may not accurately represent the fluid from mineral formed. Most of the studied samples contained volumetrically insignificant amounts of secondary inclusions, with the exception of PCS-2 and PCS-3, which exhibited appreciable amounts of secondary inclusions. Only one sample (SÜ-3; Fig. 2 and the Supplementary Material) locally contained a significant amount of the luminescent calcite; this portion of the sample was excluded from isotope analyses. Therefore, while some contribution from post-formational fluids cannot be entirely ruled out, we consider it to be insignificant for the majority of our samples.

**Fig. 6:**
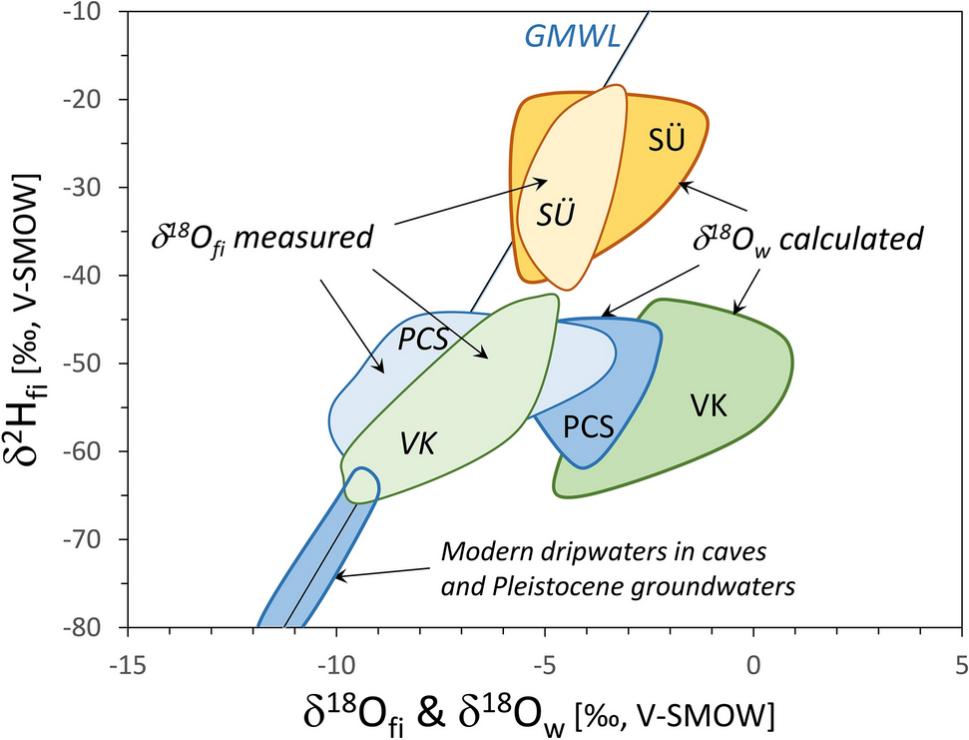
Stable hydrogen and oxygen isotope compositions of fluid inclusion waters (measured) and oxygen isotope compositions of waters calculated using calcite δ^18^O values and clumped isotope temperatures^29^ (see “Methods”). Modified after Demény et al.^29^, showing also the compositions of modern cave dripwaters^44,45^ and Pleistocene groundwaters^46^. GMWL: Global Meteoric Water Line^28^. SÜ, VK and PCS mean the locations of Sümeg, Tatabánya, and Piliscsaba, respectively.

Additional information on the contribution of post-depositional water can be obtained from the stable isotope compositions of waters. The measured δ^2^H_fi_ and δ^18^O_fi_ data plot close to the Global Meteoric Water Line (GMWL^28^) (Fig. 6). The Sümeg samples have higher δ^2^H_fi_ and δ^18^O_fi_ values and the Piliscsaba and the Tatabánya samples have overlapping lower values. Since no indications suggesting refill of fluid inclusions by post-depositional fluids were found in our samples, we interpret the stable isotope compositions of hydrogen as pristine, corresponding to the original compositions of mineral forming solutions. Additionally, Demény et al.^29^ calculated the δ^18^O values of mineral-forming waters in equilibrium with the calcites at respective T(Δ_47_) and demonstrated that the original solutions had higher oxygen isotope compositions compared to the measured δ^18^O_fi_ values (Fig. 6).

An important process that may have affected the δ^18^O_fi_ values and hence the T(OIT) calculations is the post-formational oxygen isotope exchange between the host calcite and the fluid inclusions’ water. Starting from the average δ^18^O_cc_ and T(Δ_47_) values (25 ‰ V-SMOW and 30 °C, respectively), and using the calcite-water oxygen isotope fractionation equation of Johnston et al.^5^ (1000·lnα = 17,660/–30.16, where α = (1000 + δ^18^O_calcite_)/(1000 + δ^18^O_water_) and T is temperature in K), the water composition in equilibrium with the calcite would be –3.4 ‰. Reducing the temperature to 5 °C, the water composition would be –8.6‰, fitting the δ^18^O_fi_ ranges of the Piliscsaba and Tatabánya samples in Fig. 6. Solid-state diffusion is negligible at the presumed temperature of red calcite formation^19^; thus, a dissolution and reprecipitation process is required to explain the observed T(OIT) change. This is in accordance with the observations of Uemura et al.^26^, who estimated that only 2% of the total calcite oxygen participated in the isotope exchange between the calcite and the inclusion-hosted water, even at 105 °C. This would result in the development of ~ 0.6 µm–thin alteration halos of recrystallized calcite around individual inclusion vacuoles. At lower temperatures, even smaller-scale isotope exchange would be expected, which would not affect paleotemperature estimations^26^. As such, the reason for the T(OIT) alteration observed in this study is the interplay of temperature change and time. Although the early Cenozoic, when red calcites were deposited, was likely warm, an icehouse climate developed in the Pleistocene^30^. Numerous glacial-interglacial cycles^31^ occurred during that time, so that the studied rocks could have been exposed to temperatures as low as 0–5 °C^32,33^, cumulatively for more than a million years. The slow processes of calcite dissolution, DIC-water oxygen isotope exchange, and reprecipitation may have facilitated the approach to an ca. 0 °C equilibrium in the microenvironments of fluid inclusions, shifting the δ^18^O_fi_ toward GMWL values, and T(OIT) to lower values.

Previous studies have already investigated the potential effects of retrograde isotope exchange on OIT and Δ_47_ temperatures, but these studies focused on high-temperature formations and strong cooling. Dennis and Schrag^34^ analyzed the clumped isotope compositions of carbonatites and obtained a maximum T(Δ_47_) of ~ 300 °C, whereas the crystallization temperatures are generally above 500 °C^35^. This indicates that clumped isotope compositions may also change in this temperature range by solid-state diffusion and reordering of the CaCO_3_ structure. Experimentally heating biogenic and inorganic carbonates to 130–480 °C revealed that only biogenic carbonates (mollusk shells, bivalve aragonite, belemnite rostra) suffer strong T(Δ_47_) changes, whereas inorganic calcite clumped isotope compositions remain unaffected^20,36,37^. These authors attributed the significant changes in biogenic carbonate to the effect of the large surface to mass ratio of the small inclusions present in the biogenic carbonates, which facilitates isotope exchange between the inclusion-hosted water and the host carbonate. Nooitgedacht et al.^19^ introduced a variable called the calcite exchange fraction (CEF), which is the fraction of calcite available for isotope exchange with fluid inclusions. The OIT temperatures are sensitive to retrograde isotope exchange even at low CEF, whereas the clumped isotope compositions, and therefore the T(Δ_47_), remain unaltered. Nooitgedacht et al.^19^ analyzed the oxygen isotopic compositions of calcite and inclusion-hosted water, as well as the clumped isotopic compositions in calcite veins formed in Cretaceous to Paleocene calciturbidite rocks. They obtained T(Δ_47_) temperatures of 67–86 °C and T(OIT) data of 11–59 °C (N = 4), which correspond to a T(Δ_47_)-T(OIT) difference of 53.6 ± 18.4 °C. In comparison, the Hungarian red calcite formed at approximately 30 ± 7 °C (N = 18), on the basis of the T(Δ_47_) data, resulting in a T(Δ_47_)-T(OIT) of 12.6 ± 12.9 °C. The larger T(Δ_47_)-T(OIT) difference of Nooitgedacht et al.^19^ compared to that observed for the red calcites can be attributed to the higher starting formation temperature, as all of these vein systems re-equilibrated to present-day environmental conditions and the isotope exchange durations were similar (from the Cretaceous and Paleogene until today).

These data and studies on subaqueous calcites^12,14,27^ indicate that the preservation vs. alteration of T(Δ_47_) and T(OIT) data depend on the formation temperature and the time available for isotope exchange. At low temperatures (< 100 °C), the clumped isotope data yield reliable formation temperatures, whereas the δ^18^O values of inclusion-hosted water may change^12,14,19,27^. These considerations are valid only if no late-stage fluid infiltration occurs, as recrystallization, dissolution-reprecipitation, and the formation of secondary carbonate can overwrite the original isotope signals. However, careful petrographic investigations on the calcite fabric and fluid inclusion distribution can detect, and assess their effects of the late-stage alteration processes (as presented in this study).

In summary, retrograde oxygen isotope exchange should be considered when interpreting OIT temperatures derived from the analyses of calcite and inclusion-hosted water, even if the carbonate formed at low temperatures (< 50 °C). Such exchange is facilitated by the extended time available (in this study, at least 56 million years since the Oligocene^21^) and the temperature gradient between the formation conditions (here reaching or exceeding 45 °C) and the environmental temperatures that persisted following the carbonate deposition (an annual average of approximately 10 °C at present and cooler conditions in the Pleistocene glacial periods^32^).

## Geological background and samples

Red calcite veins are ubiquitous in the Mesozoic limestones of the Transdanubian Range in western Hungary^21,22^. The sizes of the red calcite veins range from 1–2 cm to ~ 4 m in width, and the large veins display systematic zonation from dark red, fine-grained calcite at the margins to pale red and banded sparry calcite in the centers (observed in the Keselő Hill quarry^22,24^). The calcite veins examined in this study crosscut Cretaceous (in the Sintérlap quarry, Sümeg) and Triassic (in the Keselő-hegy quarry, Tatabánya and the Piliscsaba-Jászfalu quarry) limestones. The vein at the Sümeg crosscut Aptian limestone and Haas et al.^38^ described red calcite debris in Campanian-Santonian limestones, hence the red calcite dike formed most likely during the late Cretaceous. By studying several red calcite locations in the Transdanubian Range, Western Hungary, Győri et al.^21^ suggested that red calcite formed from the Triassic to the Oligocene. More precise age determinations of the red calcite veins have not been performed.

To determine the formation conditions of red calcite, Demény et al.^22^ employed fluid inclusion microthermometry and calculations involving stable isotope analyses of calcite and inclusion-hosted water. On the basis of homogenization temperatures ranging from 100 to 190 °C, Demény et al.^22^ calculated the stable isotopic compositions of mineral-forming water and interpreted the formation of calcite veins as hydrothermal fracture fillings that were induced by fluid migration related to magmatic activity. This interpretation was questioned by Győri et al.^21^, who, on the basis of fluid inclusion petrography, suggested that the high homogenization temperatures reported by Demény et al.^22^ were either related to poor preservation of fluid inclusion volume (stretching and/or leakage) or were a result of heterogeneous fluid entrapment. They interpreted the red calcite occurrences as speleothems formed at low temperatures (< 50 °C) from descending solutions that infiltrated through bauxite (hence the calcite’s red color).

The samples researched in this study were collected at three locations: Sümeg’s Sintérlap quarry, Tatabánya’s Keselő-hegy quarry, and Piliscsaba’s Piliscsaba-Jászfalu quarry (Fig. 1). In the Tatabánya and Piliscsaba locations, large (50–100 cm) debris blocks were sampled that were taken from the walls (Fig. 1), whereas in the Sümeg location, the red calcite vein was sampled in situ. The ~ 4 m wide red calcite vein^22^ was consumed due to the quarry’s operation. At the Sümeg location, samples with pale and dark banding (SÜ-1 and SÜ-2), as well as dark red calcite masses (SÜ-3) were collected, in addition to the limestone-vein contact (SÜ-4). In the Tatabánya quarry, two blocks were sampled, yielding dark red calcites (VK-1 and VK-6) and pale red calcites (VK-2 and VK-4). The block sampled in the Piliscsaba quarry yielded dark red calcites (PCS-1) and lighter red, sparitic calcites (PCS-2 and PCS-3). The specimens were subsampled to detect internal inhomogeneities. The Supplementary Material contains photos of the samples.

## Methods

Observations and documentation of calcite fabric and fluid inclusion petrography were performed using a Nikon Eclipse optical microscope at the Institute of Geology, Innsbruck University, Innsbruck. Observations were made on doubly polished thick sections (ca. 100 µm). The fluid inclusion characteristics (primary vs. pseudosecondary and secondary) were determined based on petrographic observations. The primary origin was inferred on the basis of (a) a clear association of fluid inclusion assemblages (FIAs^39^) with growth-defined features (growth zones, former growth surfaces, compromise growth boundaries, etc.) and of (b) the specific morphology of individual fluid inclusion vacuoles, such as flared, tapered, waisted, and flat-bottomed. The specific shapes of these inclusions (which are provisionally called ”directional inclusions”) reflect the direction in which the host calcite grew, unambiguously indicating the primary character of the inclusions. Cathodoluminescence microscopic pictures were taken with a Reliotron “cold-cathode” instrument mounted on a Nikon Eclipse E600 microscope with a Nikon Coolpix 4500 digital camera, operated at 6 to 10 keV acceleration voltage.

The stable hydrogen and oxygen isotopic compositions of the inclusion-hosted water were determined at the Institute for Geological and Geochemical Research in Budapest, following Demény et al.^13^. Sample chips (2–5 mm pieces) of approximately 1–2 g were crushed under vacuum in 10 mm (outer diameter) stainless steel tubes, and the extracted H_2_O was purified by vacuum distillation to remove non-condensable gases and CO_2_. H_2_O, was introduced into a model LWIA-24d (Los Gatos Research Ltd.) liquid water isotope analyzer. The amount of H_2_O was calibrated by injecting known amounts of water with a syringe. Corrections for measurement drifts and amount and memory effects were conducted using three laboratory water standards^40^. The isotopic compositions are expressed in standard *δ* notation in ‰ relative to V-SMOW. The estimated analytical accuracies are ca. ± 0.5 and ± 2‰^32^ for *δ*^18^O_fi_ and *δ*^2^H_fi_ (where “fi” means “fluid inclusion”), respectively, based on measurements of carbonate-hosted inclusion waters with known isotopic compositions.

Clumped, as well as stable carbon and oxygen isotope analyses of carbonates were carried out at the Isotope Climatology and Environmental Research Centre (ICER), Institute for Nuclear Research (ATOMKI) in Debrecen, Hungary. After phosphoric acid digestion at 70 °C, carbonate sample analysis was performed on a Thermo Scientific™ 253 Plus 10 kV isotope ratio mass spectrometer (IRMS) using a Thermo Scientific Kiel IV automatic carbonate device. All of the measurement data, including sample and standard measurements, are listed in the Supplementary Tables. The international reference sample IAEA C-2 was measured as unknown and yielded δ^13^C = –8.28 ± 0.05 ‰ and δ^18^O = –9.05 ± 0.10 ‰ (n = 195), whereas δ^13^C = –8.25 ± 0.02 ‰ and δ^18^O = –9.00 ± 0.05 ‰ (n = 11) were reported for the ETH Zürich laboratory^41^, thereby demonstrating the accuracy of the isotopic data. The clumped isotope analyses were standardized using the ETH-1,2,3 carbonate standards^41^^,.^^42^. The condition of the PoraPak™ Q trap was monitored through the Δ_49_ WG (PBL) values in the replicates of the ETH1 standard. Moreover, the potential of organic contamination in the extracted gas was assessed by examining the measured Δ_48_ and Δ_49_ values for each replicate. Further details are provided by Demény et al.^12^.

Temperatures were calculated using the measured oxygen isotope fractionation between calcite and inclusion-hosted water, as well as with the fractionation-temperature equation of Johnston et al.^5^. The actual equation that exactly describes the studied calcite-water system is not known. Following the approach of Demény et al.^12^ to calculate the temperatures using the most distant equations^1,6^, and to use the averages is problematic in our case, as the equation of Chacko and Deines yielded unrealistically low (< 0 °C) temperatures, and the environments on which the Daëron et al.^6^ equation is based (extremely slowly precipitating calcites in closed environments) may not be considered analogous to the open cracks in which the red calcites formed. Instead of using the equations that would yield the most distant temperature values, the equation of Johnston et al.^5^ was selected, which is based on a compilation of natural speleothem data. The propagated errors of the calculated temperatures are based on the uncertainties of the equation^5^ (~ ± 0.05 in the 1000/T values estimated using their Fig. 6), and the uncertainties of δ^18^O measurements of calcites and fluid inclusion waters (0.1 and 0.5 ‰, respectively). The clumped isotope temperatures were calculated following the calibration of Anderson et al.^17^ and the D47crunch algorithm^43^. The uncertainties of the calculated temperatures are given as 95% confidence intervals (95% CI) in Table 1, but standard errors are also listed in the Supplementary Tables.

## Supplementary Information

Supplementary Information 1.

Supplementary Information 2.

Supplementary Information 3.

Supplementary Information 4.

## Data Availability

All data generated or analysed during this study are included in this published article [and its supplementary information files].
