# Supplementary material for: Preservation and alteration of inclusion-based calcite-water oxygen isotope and clumped isotope temperature signals in calcite veins
Source: Sci Rep. 2025 Mar 14;15:8800. doi: 10.1038/s41598-025-92824-w (PMC11909253; doi:10.1038/s41598-025-92824-w)
Supplement: Supplementary file 1 — Supplementary Information 1. [file 41598_2025_92824_MOESM1_ESM.pdf]

Preservation and alteration of inclusion-based calcite-water oxygen isotope and clumped isotope temperature signals in calcite veins

Attila Demény[1,2], László Rinyu[3], Yuri Dublyansky[4], Bernadett Bajnóczi[1,2]

[1] Institute for Geological and Geochemical Research, HUN-REN Research Centre for Astronomy and Earth Sciences, Budapest, Budaörsi út 45, H-1112, Hungary

[2] CSFK, MTA Centre of Excellence, Budapest, Konkoly Thege Miklós út 15-17., H-1121, Hungary

[3] Isotope Climatology and Environmental Research Centre, HUN-REN Institute for Nuclear Research, Debrecen, Bem tér 18/C, H- 4026, Hungary

[4] Institute of Geology, University of Innsbruck, Innrain 52, 6020 Innsbruck, Austria

Supplementary Material

Contents

- Photos of red calcite samples

- Petrographic analysis of red calcite samples

Photos of red calcite samples

Piliscsaba, Piliscsaba-Jászfalu quarry.

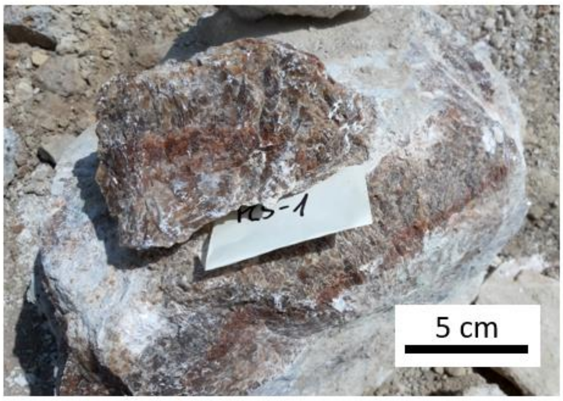

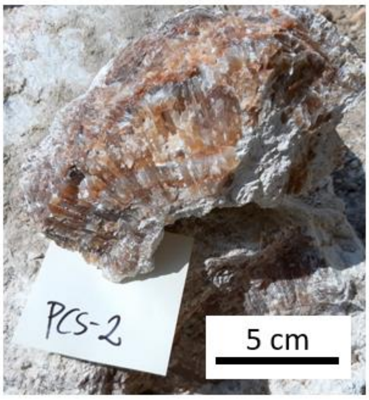

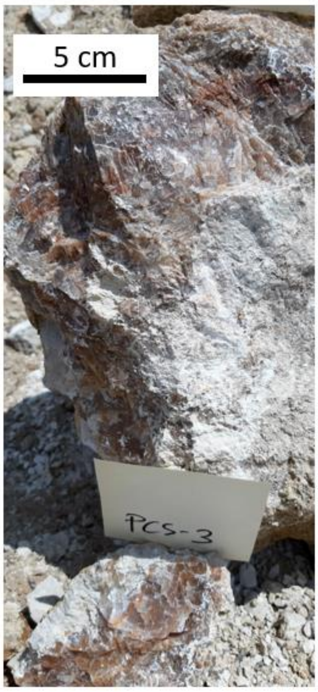

Tatabánya, Keselő-hegy quarry

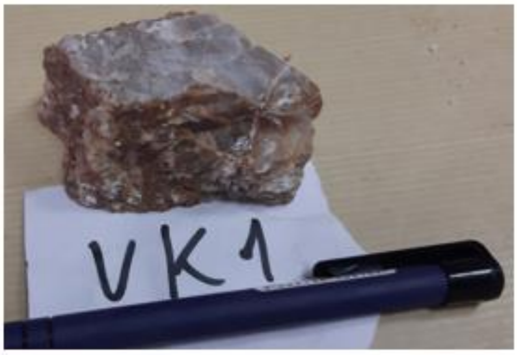

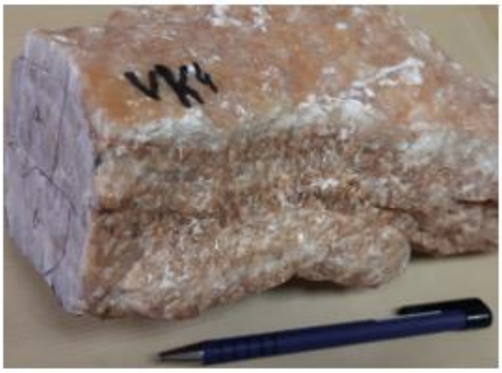

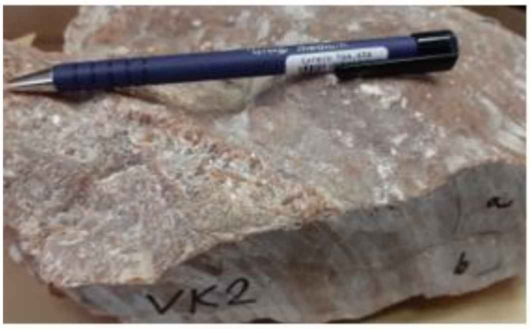

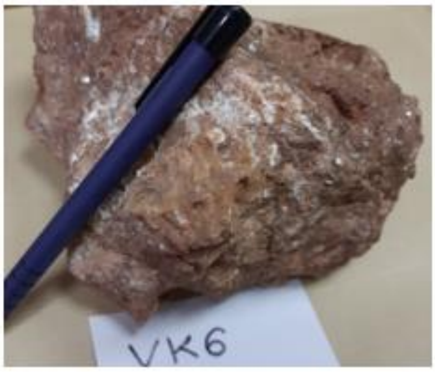

Sümeg, Sintérláp quarry

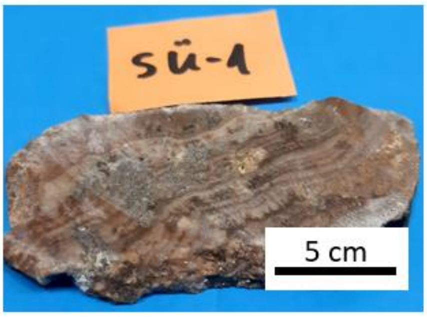

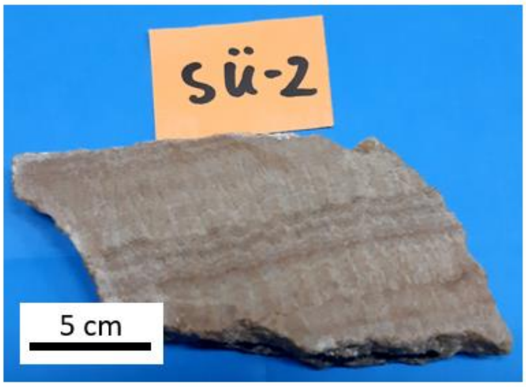

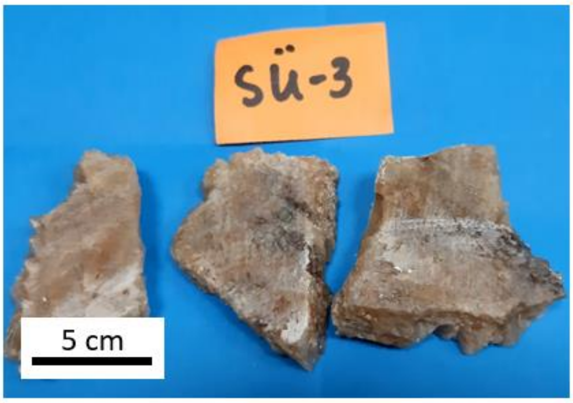

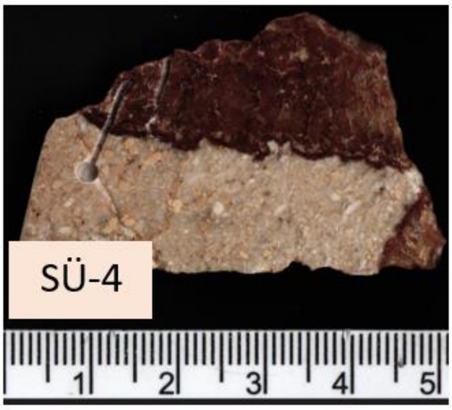

Petrographic analysis of red calcite samples

Sample PCS-1

Calcite fabric

Calcite has a characteristic fabric, featuring elongate columnar crystals bounded by compromise growth boundaries (compact columnar fabric of Frisia et al., 2000; Fig. 1a). Crystals are arranged in 'bundles' with slightly different optical orientation (Fig. 1a). Growth zones, particularly clear in upper part of the sample, transcend these 'bundles' and optical domains.

There is a prominent, but local, break in deposition of calcite, likely caused by contamination of the growth surface by some mineral material (lower red arrow in Fig. 1a). The formed 'gap' was later filled with younger layers of calcite. The younger calcite shows indications of mechanical damage (twin lamellae; yellow arrow in Fig. 1b). Interestingly, older calcite does not show traces of twinning.

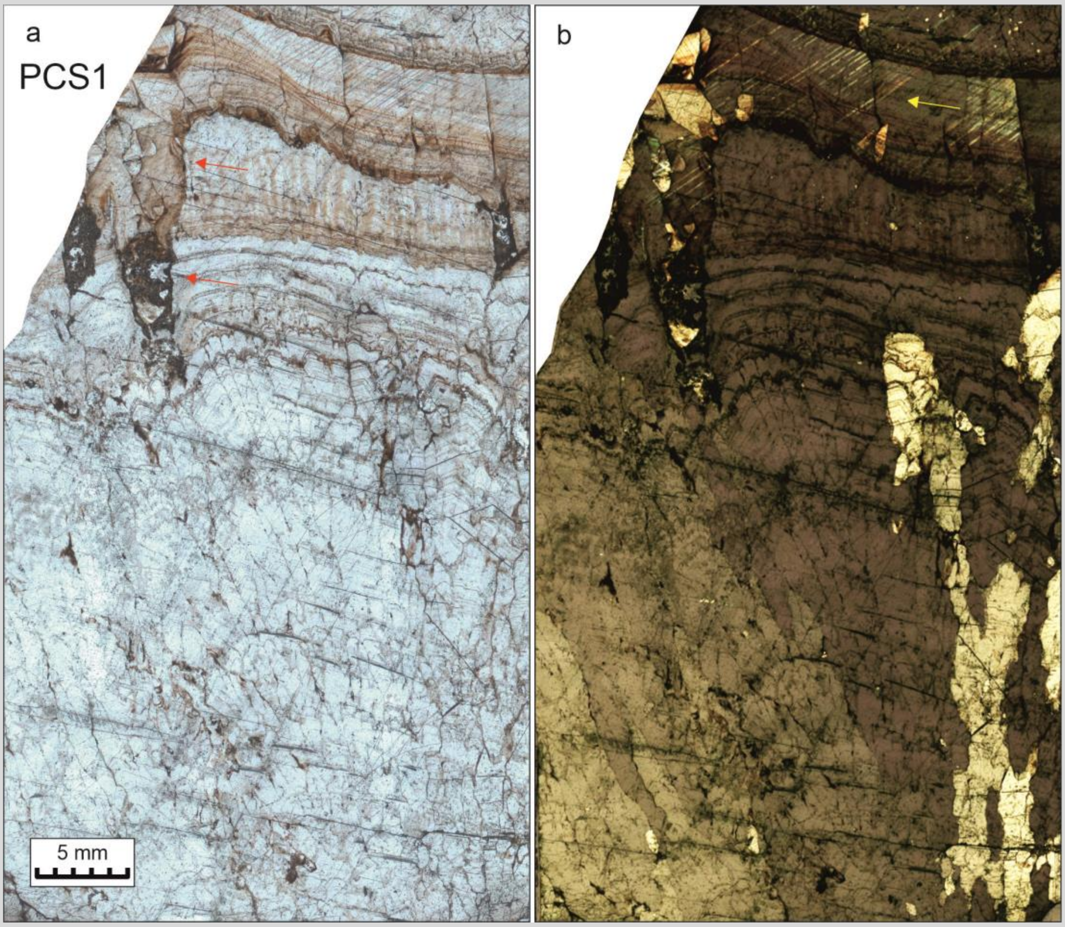

Fig. 1. Section PCS-1: (a) plane-parallel light; (b) polarized light. Local break in deposition is marked by red arrows in **a**; yellow arrow in **b** points to mechanical twins.

Filaments

Filaments were observed in calcite of the sample (Fig. 2). They are not abundant, but persistently present. These filaments are interpreted as biological remnants (filamentous bacteria or fungi) entrapped by growing calcite.

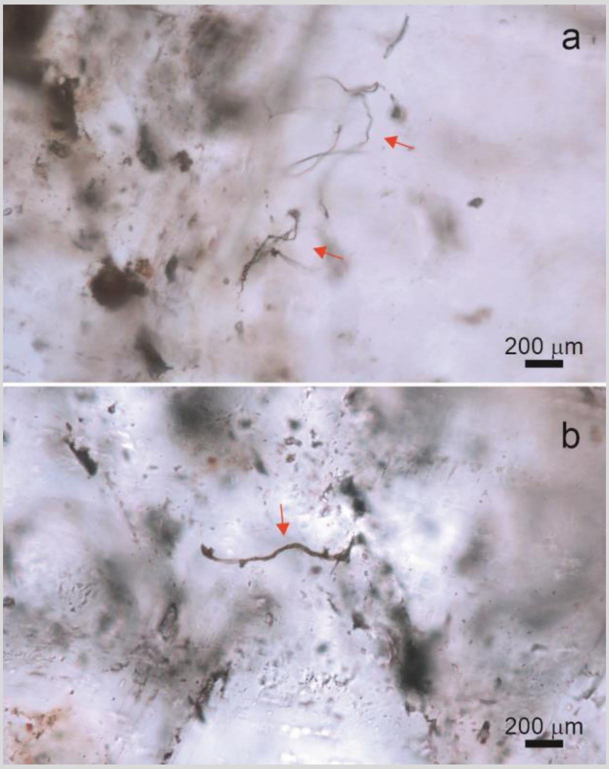

Fig. 2. Section PCS-1; filaments embedded in calcite (red arrows): (a) group of filaments; (b) individual filament; calcite around it is slightly dissolved, producing a tubular vacuole. Filaments could be remnants of filamentous bacteria or fungi.

Inclusions

Fluid inclusions are abundant in this sample. Morphologies of inclusions vary from highly irregular to simple, nearly isometric.

Most of fluid inclusions in this sample can be classed as primary. This is inference is supported by: (a) clear association of fluid inclusion assemblages (FIAs, Goldstein and Reynolds, 1994) with growth-defined features (growth zones, former growth surfaces, former compromise growth boundaries); and (b) specific morphology of individual fluid inclusion vacuoles (directional inclusions, whose shape is controlled by the direction of growth of the host calcite crystals or subcrystals; flared; tapered; waisted; flat-bottomed). This feature allows unequivocally infer their primary character Examples are shown in Figs. 3 and 4.

Most of the inclusions in this sample are single-phase liquid (L), aqueous. Yet, a relatively significant number of inclusions (roughly, about 1 %), contain vapor bubbles (Fig. 4). The vapor-to-liquid (V/L) ratios are (on visual basis) about 0.1–0.2.

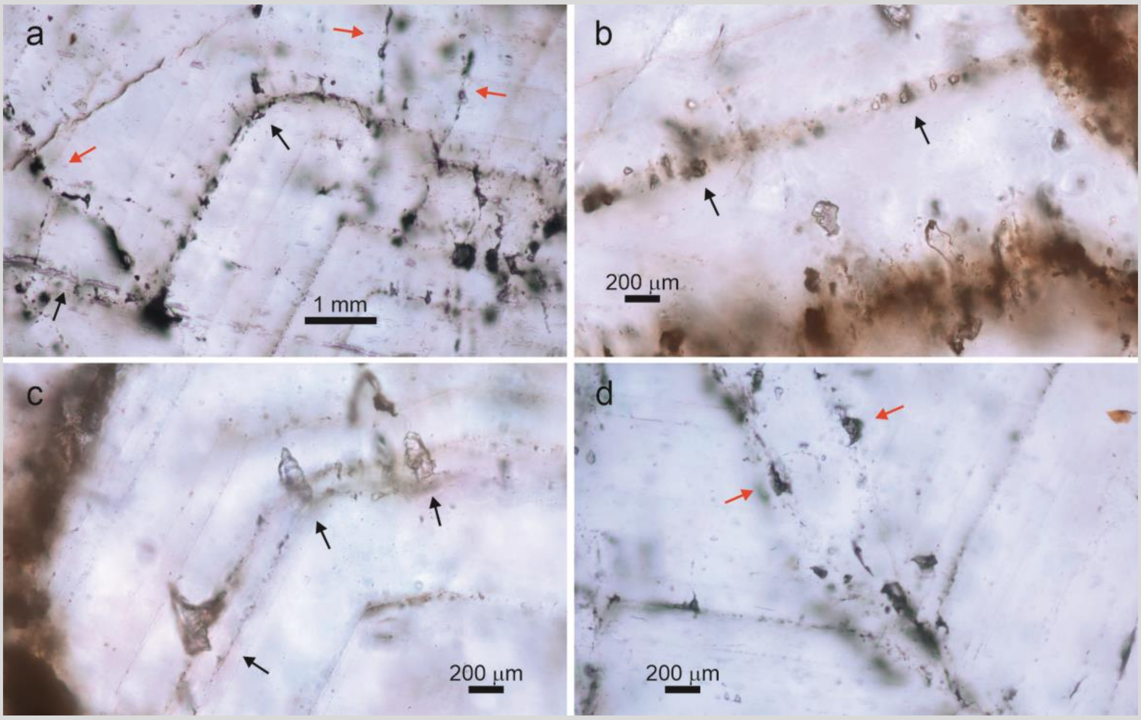

Fig. 3. Section PCS-1: (a) inclusions associated with growth zones (black arrows) and with compromise growth boundaries (red arrows); (b, c) inclusions associated with growth zones; (d) inclusions associated with compromise growth boundaries. Most of inclusions are single-phase liquid, aqueous.

Overall interpretation of PCS-1

Calcite. The character of calcite (fabrics) strongly suggests its speleothemic origin[1]. At some point in time the calcite has experienced stress, which resulted in mechanical twinning of the youngest part of calcite exposed in this section.

Fluid inclusions. Most of inclusions in this sample are primary, although some amounts of secondary inclusions cannot be precluded. Both intra-crystalline and inter-crystalline inclusions are present.

Traces of biological activity. Filaments, representing remnants of contemporaneous life (likely, fungi) are entrapped in calcite. This is consistent with relatively low-temperature environment of calcite formation.

Temperature of formation. Based on the available evidence, calcite formed at relatively low temperature.

After its formation, this calcite has likely been exposed to some elevated-temperature conditions, which led to stretching of some of the originally single-phase liquid inclusions. This caused the appearance of vapor bubbles in some of the inclusions. Mechanical damage of calcite could also lead to the appearance of bubbles in originally single-phase aqueous inclusions; in this case, however, the V/L ratios are expected to be highly variable, which is not observed in this sample.

[1] The term 'speleothemic' is used to define deposition of calcite in open cavities, which could be of karstic or non-karstic origin (e.g., open tectonic fractures).

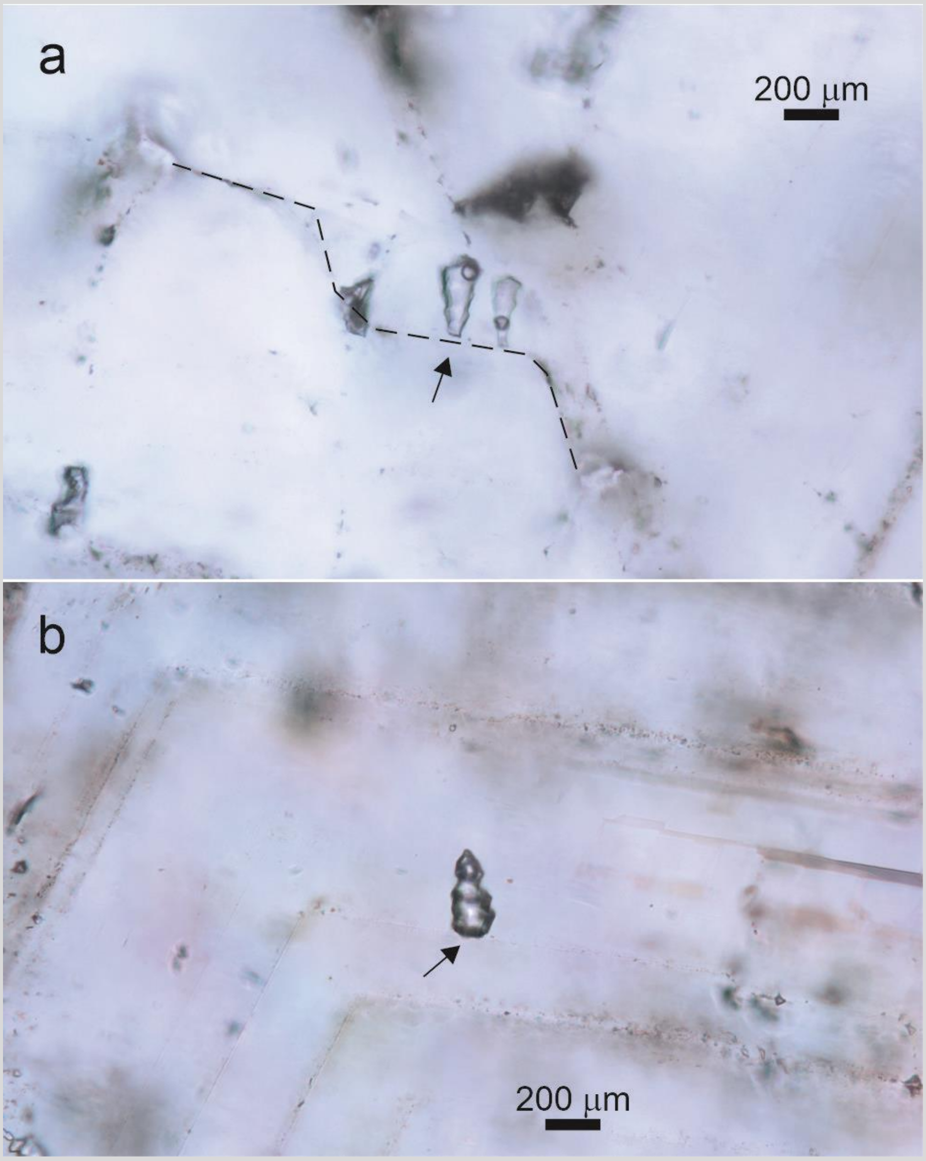

Fig. 4. Section PCS-1; inclusions, originating at former growth surfaces (black arrows): (a) two two-phase (LV) inclusions; somewhat consistent V/L ratio; (b) waisted-shape LV inclusion.

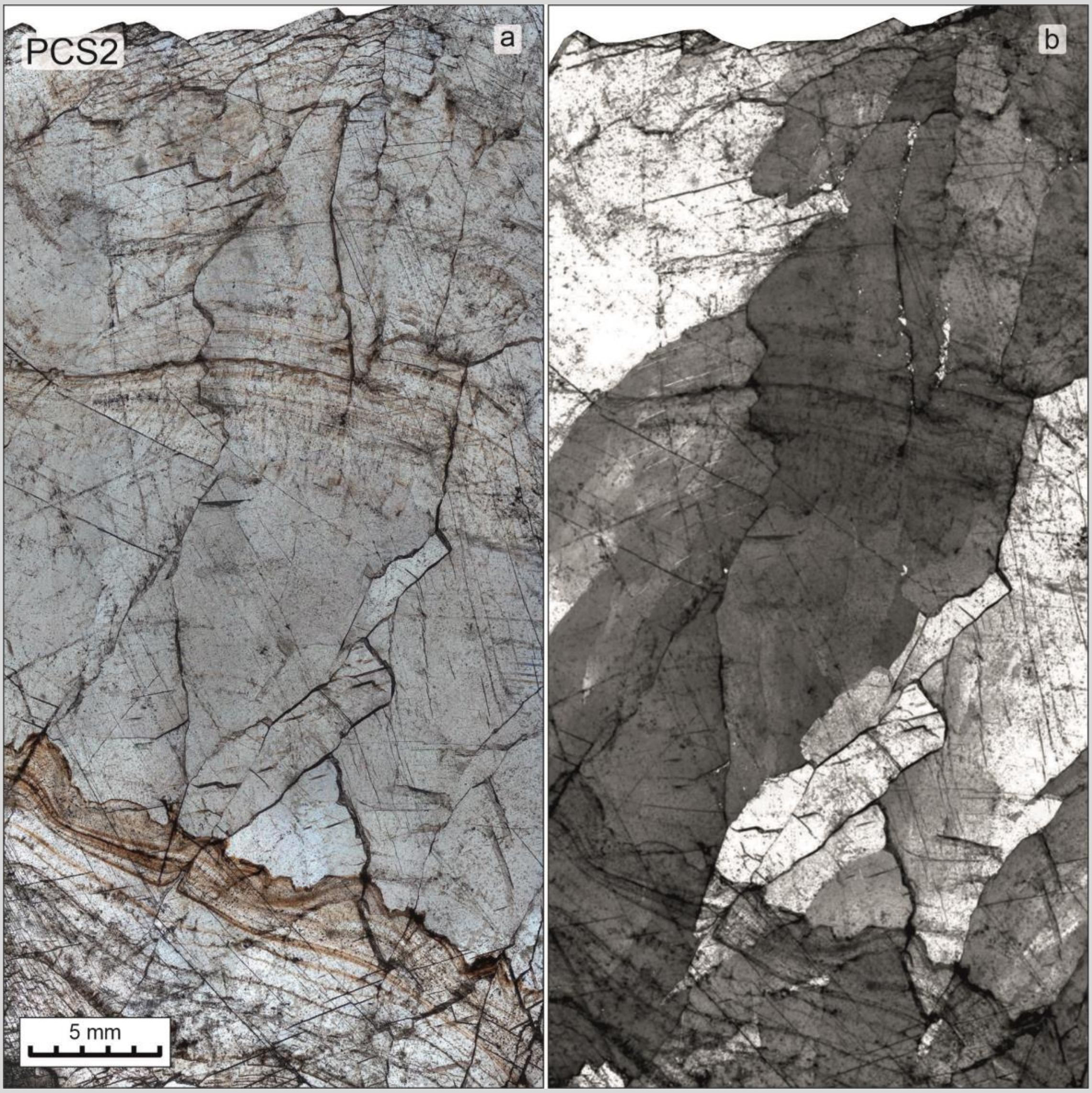

Fig. 5. Section PCS-2: (a) plane-parallel light; (b) polarized light.

Sample PCS-2

Calcite fabric

Petrographic characteristics of calcite in this sample are similar to sample PCS-1. It has a characteristic compact columnar fabric (Fig. 5).

Filaments

Similarly to sample PCS-1, filaments were observed in calcite of this sample (Fig. 6).

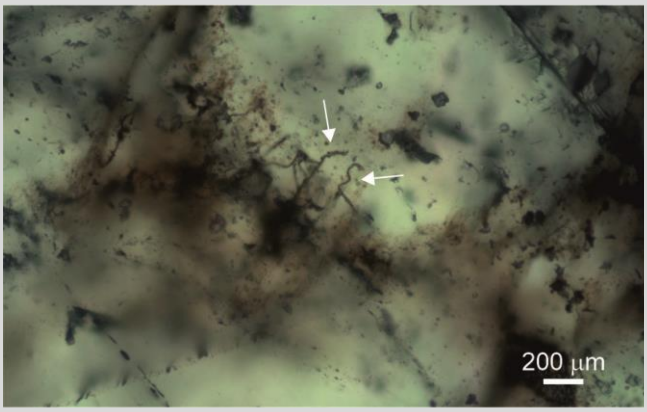

Fig. 6. Section PCS-2: Filaments embedded in calcite (white arrows). Filaments could be remnants of filamentous bacteria or fungi.

Inclusions

Most of inclusions are primary (directional), distributed 3-dimensionally in the calcite matrix (Fig. 7a). Sometimes inclusions are more concentrated along growth zones (Fig. 7b).

In this sample, inclusions also occur along fractures (that is, secondary inclusions). Such inclusions are sometimes much larger than primary ones, and have, typically, very irregular shapes (Fig. 8). Although such secondary FIAs are not common, the volume of water contained therein can be large.

Overall interpretation of PCS-2

Interpretation of calcite fabrics, fluid inclusions, and temperature of formation for this sample is identical to sample PCS-1.

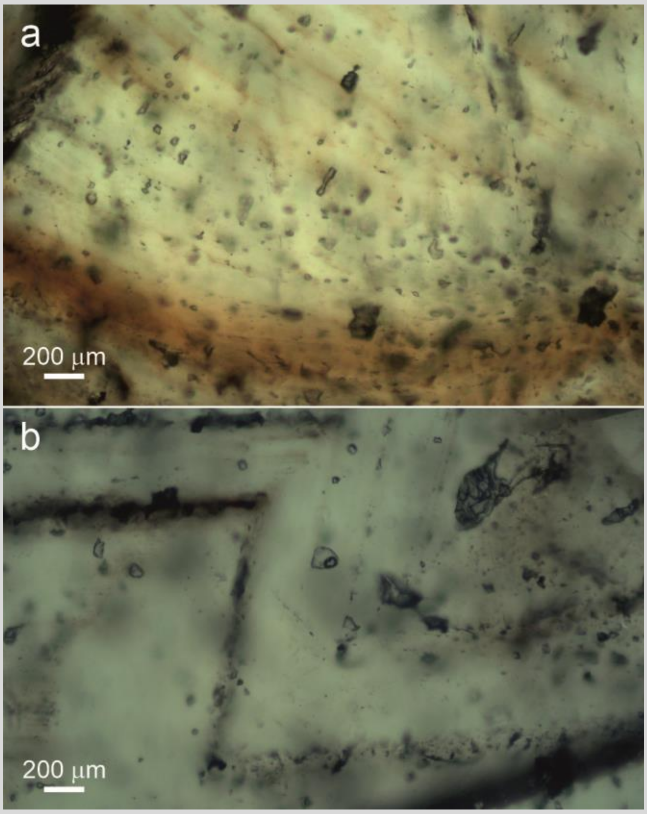

Fig. 7. Section PCS-2: (a) primary inclusions, forming a 3D 'cloud', in which individual inclusions are arranged along the former crystal growth surfaces; (b) primary flat-base LV inclusion, originating at former crystal face.

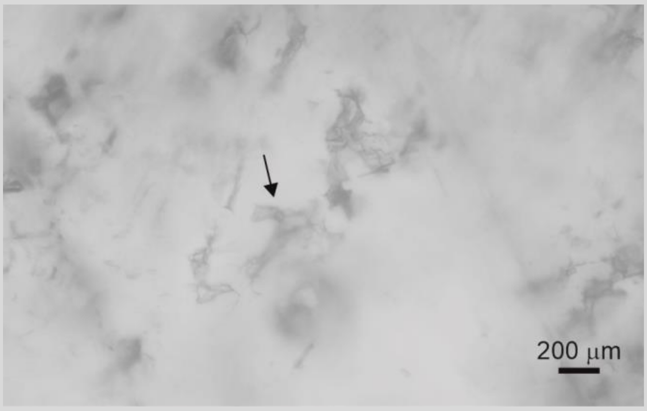

Fig. 8. Section PCS-2: Large irregularly-shaped secondary inclusions along cross-cutting fracture. One of the inclusions contains vapor bubble (arrow).

Sample PCS-3

Calcite fabric

Petrographic characteristics of calcite in this sample are similar to samples PCS-1 and PCS-2. It has a characteristic compact columnar fabric (Fig. 9). Traces of moderate mechanical twinning can be observed.

Filaments

None observed in this sample.

Inclusions

Most of inclusions in this sample are primary. They are aligned along growth zones (Fig. 10 a, b), or form 3D clusters in crystal cores (Fig. 10 c). Spatial distribution of inclusions is irregular; highly concentrated in some places and nearly absent in other (cf. Fig 10 a-c). Importantly, among primary inclusions there are single-phase vapor ones (they appear dark under microscope). This indicates that this spelothemic calcite was formed, most likely, subaerially (flowstone).

Inclusions are also present along cleavage fractures; these inclusions are, most likely, secondary.

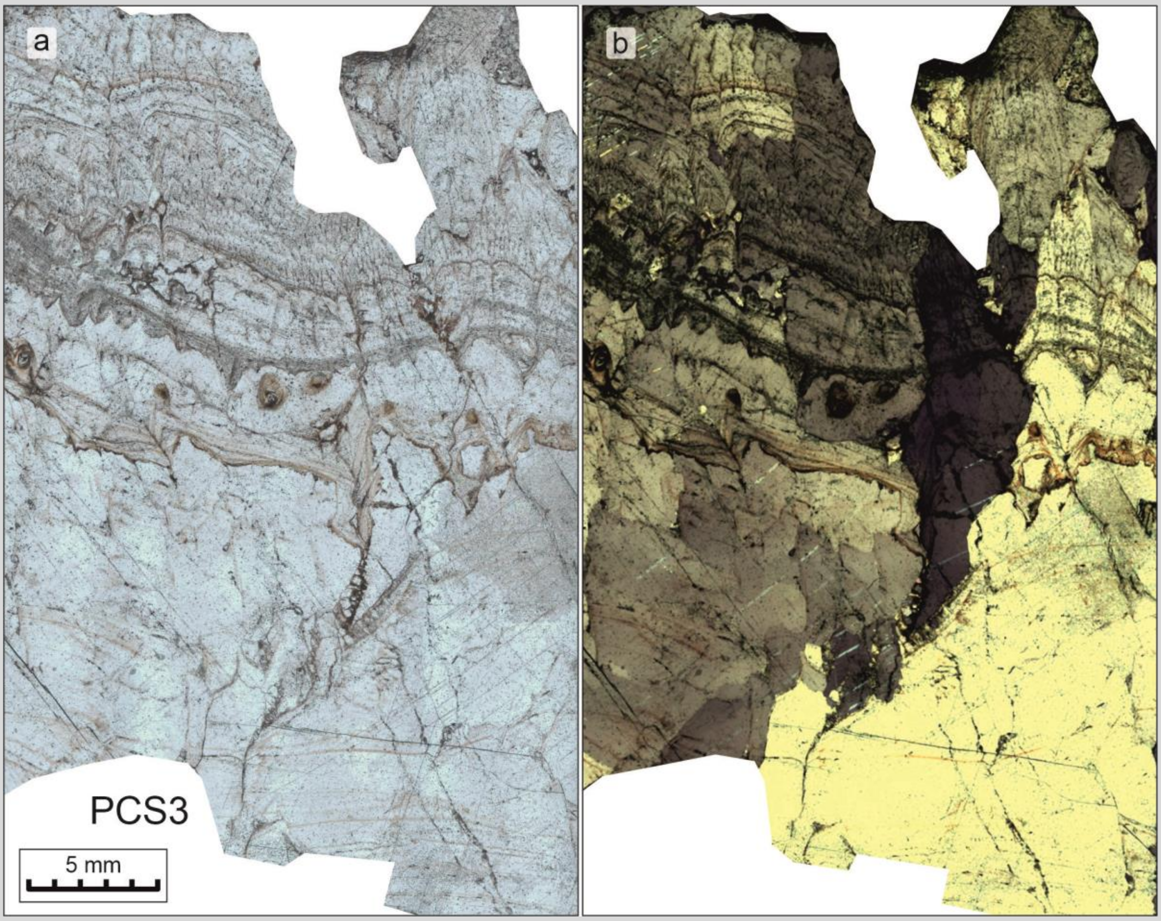

Fig. 9. Section PCS-3: (a) plane-parallel light; (b) polarized light.

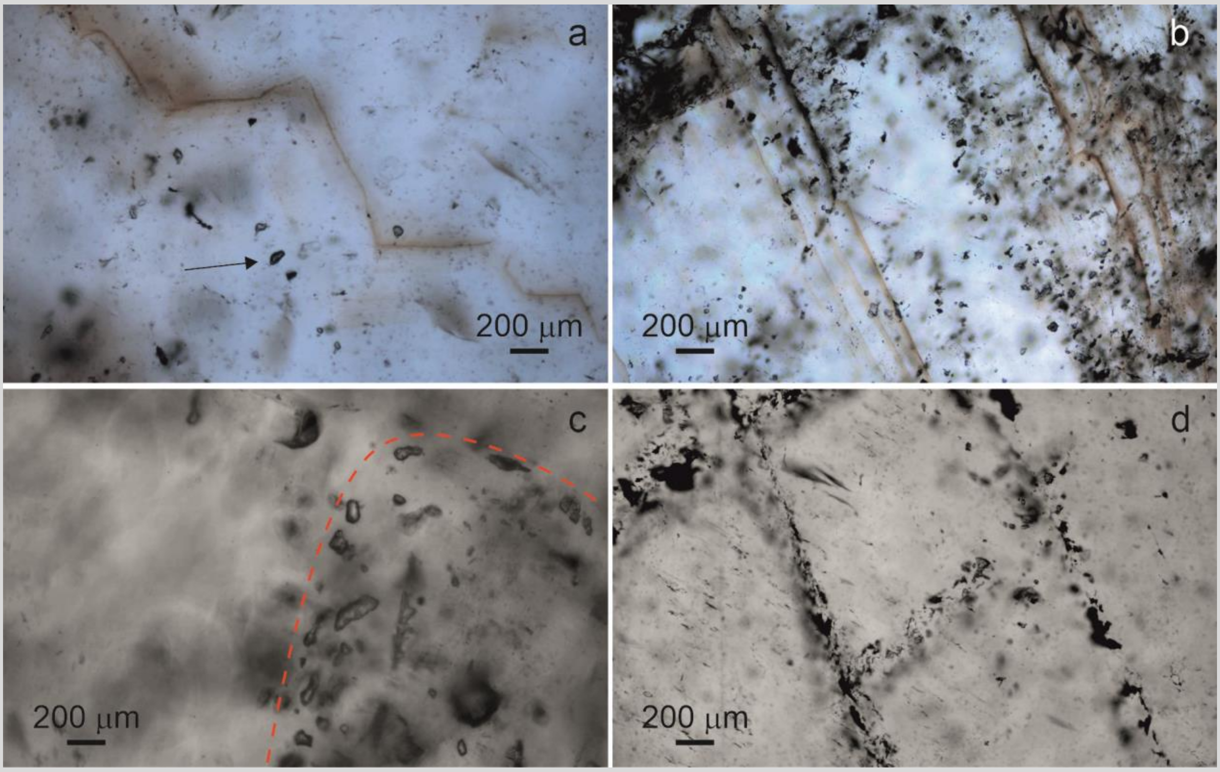

Fig. 10. Section PCS-3: (a) scarce inclusions, associated with former growth surfaces; note single-phase vapor (V) inclusion (arrow); (b) numerous small inclusions associated with former growth surfaces; (c) 3D 'cloud' of relatively large aqueous inclusions in the core of the crystal; (d) numerous small secondary inclusions trapped along cleavage fractures.

Overall interpretation of PCS-3

Calcite. Similar to PCS-1 and PCS-2.

Fluid inclusions. The presence of primary single-phase vapor (V) inclusions is consistent with vadose (flowstone) origin.

Temperature of formation. Based on exclusively single-phase character of primary inclusions, the host calcite was formed at relatively low temperatures (less than *approximately* 40°C).

Sample SÜ-1

Calcite fabric

Calcite has a fabric, built up of elongate columnar crystals (open columnar fabric of Frisia et al., 2000; Fig. 11a). In places, adjacent crystals (or small bundles of crystals) do not coalesce, and space between them either remain empty or gets filled with dark material. In other domains, crystals do coalesce, and form compromise growth boundaries. Some domains have ‘through’ optical orientation, whereas in other domains adjacent crystals are variously oriented (Fig. 11b).

Growth zones, transcend ‘bundles’ in domains with continuous calcite deposition and become less pronounced where inter-crystalline porosity is abundant.

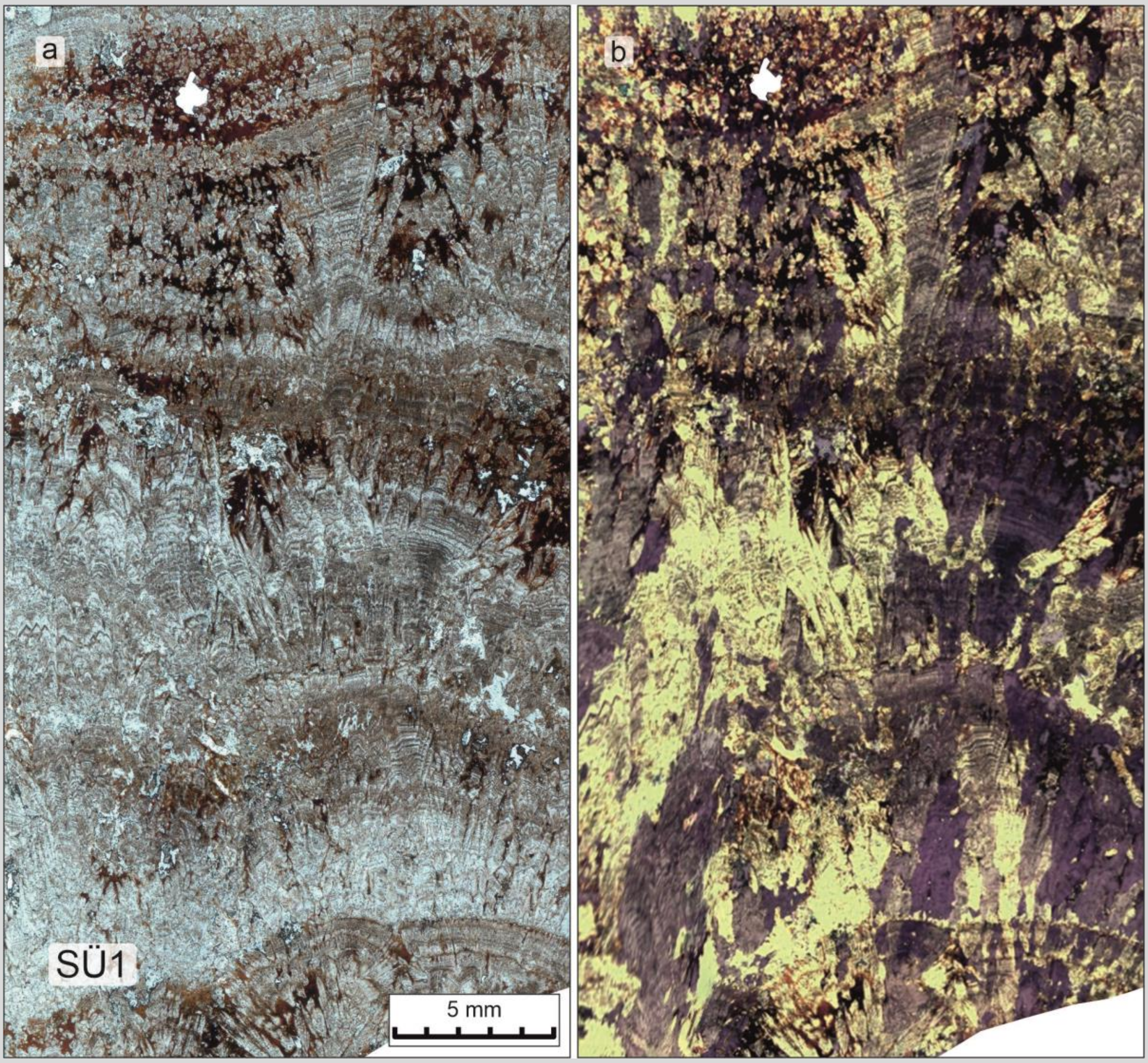

Fig. 11. Section SÜ-1: (a) plane-parallel light; (b) polarized light.

Inclusions

Fluid inclusions are abundant. Morphologies of inclusions are predominantly elongate, irregular spindle-shaped, with aspect ratios ranging from 2–3 to 20–30.

Orientation of inclusions relative to growth zones (Fig. 12) testifies for their primary character; primary inclusions absolutely dominate the sample. Almost all inclusions are single-phase liquid, aqueous (accidental vapor bubbles were observed in less than 0.01% of inclusions). Some dark (air-filled) inclusions are interpreted as leaked (most likely, during sample preparation).

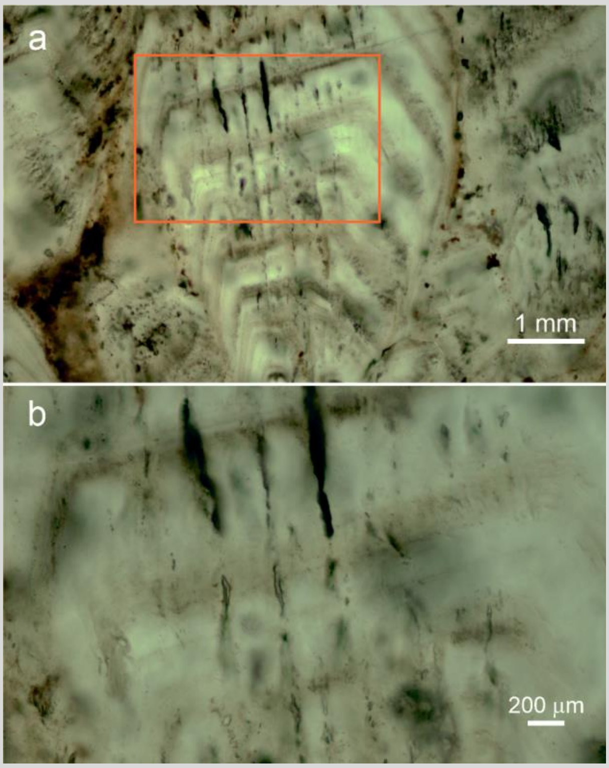

Fig. 12. Section SÜ-1: (a) elongate single-phase liquid (L) inclusions, transcending the former crystal growth surfaces; (b) close-up.

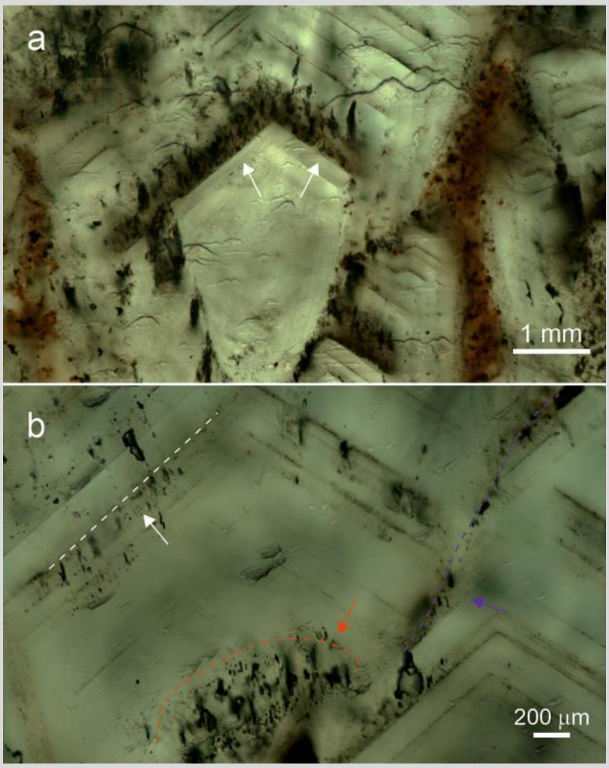

Fig. 13. Section SÜ _1: (a) densely spaced inclusions, originating at former crystal growth surface (white arrows); (b) inclusions associated with growth surfaces (white arrow and dashed line), inclusions forming a 2D 'cloud' at the core of the crystals (red arrow and dashed line), and inclusions trapped along compromise crystal growth boundary (blue arrow and dashed line).

Characteristic occurrences of fluid inclusions comprise: (a) FIAs occurring as 3D clouds at crystal cores (Fig. 13b); (b) disperse inclusions oriented normally to growth zones (either starting from zones, or transcending them; Fig. 12 and 13); (c) inclusions trapped along compromise growth boundaries between crystals or their bundles (Fig. 13b). This implies that the sample contains both intra- and inter-crystalline inclusions.

Overall interpretation of SÜ-1

Calcite. The character of calcite (fabrics) strongly suggests its low temperature origin.

Fluid inclusions. Most of inclusions in this sample are primary, although some (minor) amounts of secondary inclusions cannot be precluded. Both intra- crystalline and inter-crystalline inclusions are present.

Temperature of formation. Based on the sum of evidence, calcite formed at relatively low temperature (less than *approximately* 40°C).

Sample SÜ-2

Calcite fabric

Calcite has elongated columnar fabric with clearly defined, sharp growth zones commonly emphasizing multiple euhedral crystal terminations of the advancing growth surfaces (Fig. 14).

Inclusions

Character of fluid inclusions in this sample is virtually identical to sample SÜ-1.

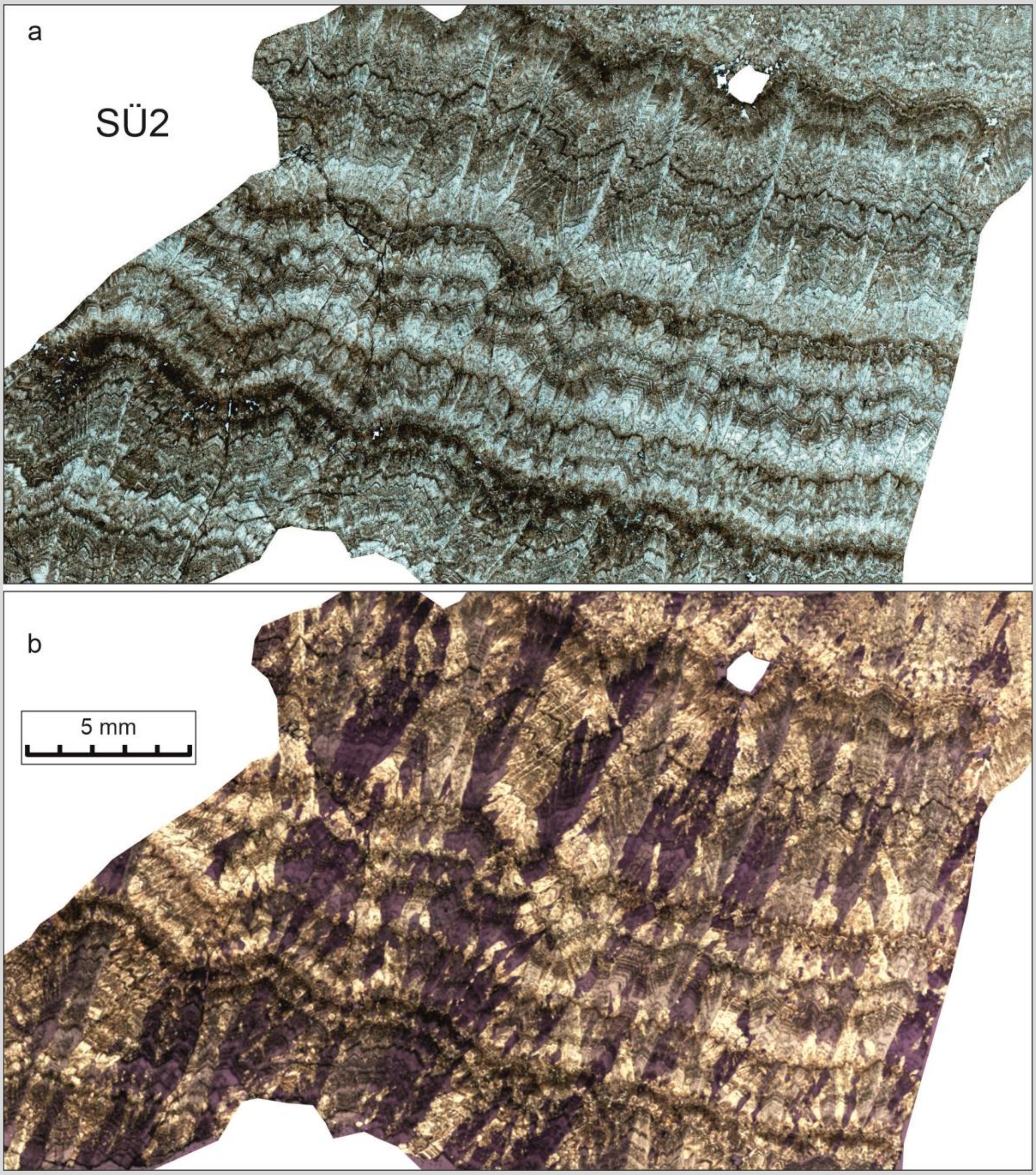

Fig. 14. Section SÜ-2: (a) plane-parallel light; (b) polarized light.

Fluid inclusions are abundant. Morphologies of inclusions are predominantly elongate, irregular spindle-shaped, with aspect ratios ranging from 2–3 to 20–30 (Fig. 15).

Overall interpretation of SÜ-2

Calcite. The character of calcite (fabrics) is compatible with its low temperature origin.

Fluid inclusions. Most of inclusions in this sample are primary. Both intra- and inter-crystalline inclusions are present; intra-crystalline dominate.

Temperature of formation. Based on fluid inclusion evidence, calcite has formed at relatively low temperature (less than *approximately* 40°C).

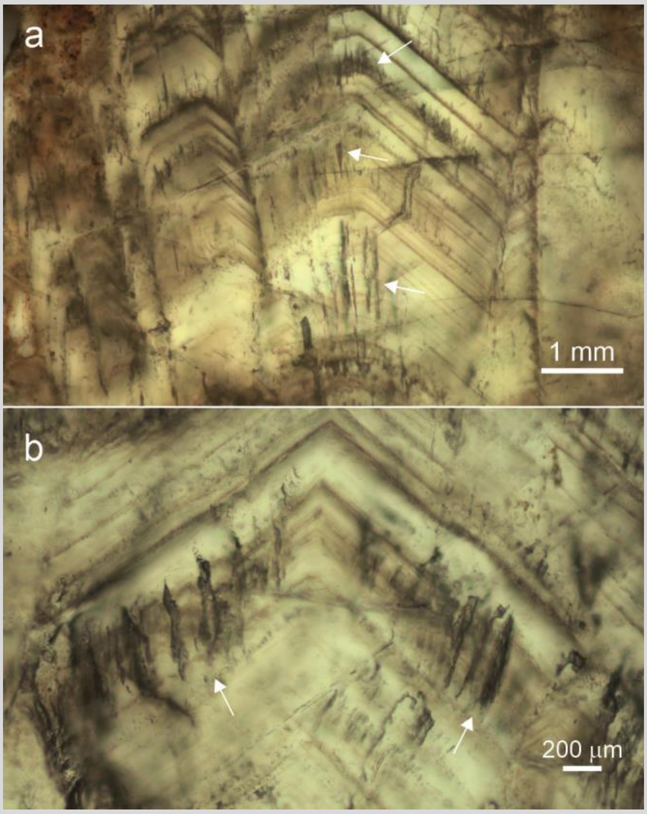

Fig. 15. Section SÜ-2: (a) elongate single-phase liquid (L) inclusions, transcending the former crystal growth surfaces; (b) close-up.

Sample SÜ-3

Calcite fabric

Calcite in this sample is dissimilar from samples SÜ-1 and SÜ-2 (Fig. 16). It consists of large columnar crystals, featuring tight compromise growth boundaries. The crystals have only slightly different optical orientation (Fig. 16b).

The calcite was affected by mechanical stress and shown at least three sets of twin lamellae (Fig. 16). Mechanical stress, however, did not lead to deformation of fluid inclusions sufficient to produce bubbles.

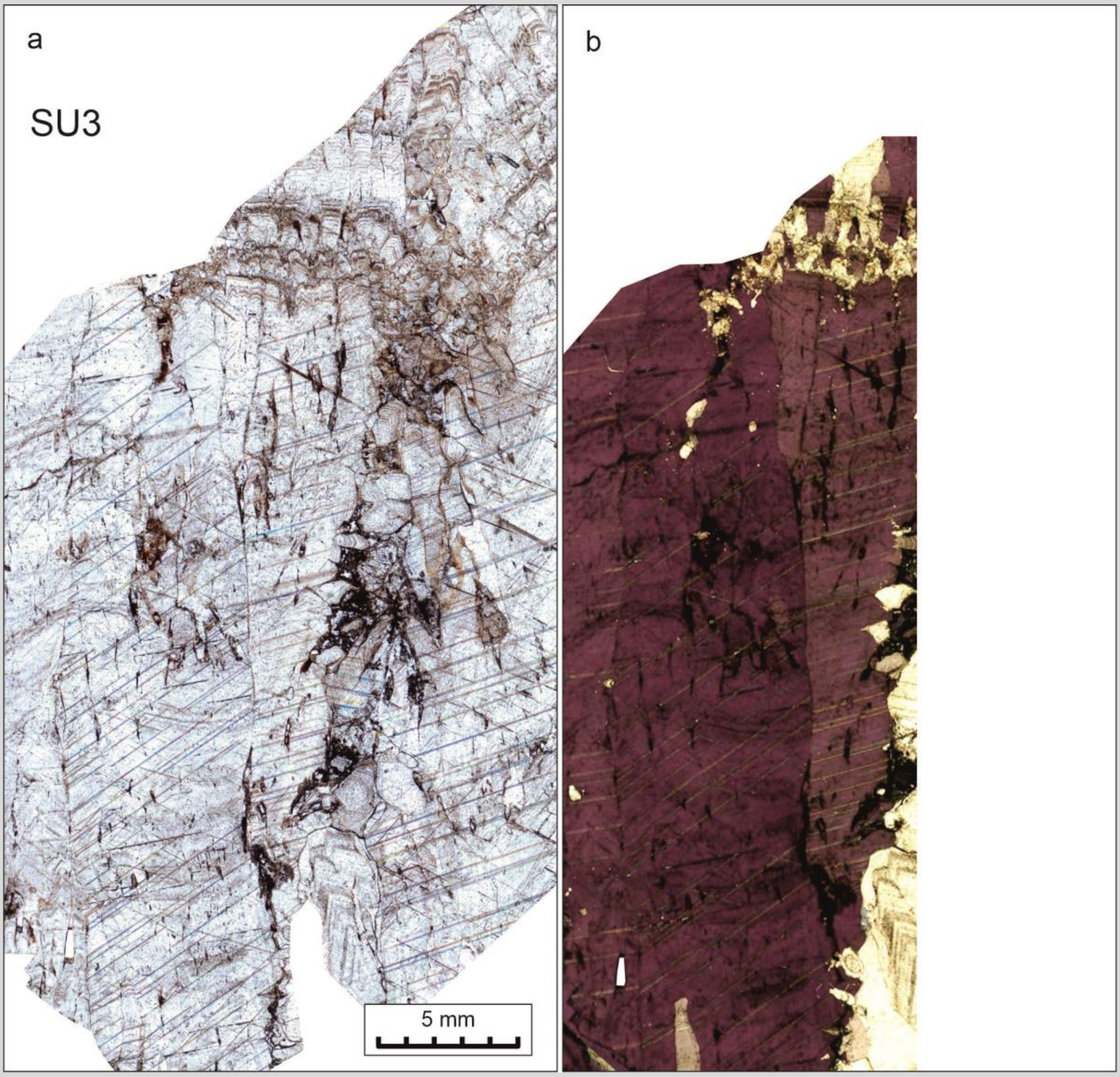

Fig. 16. Section SÜ-3: (a) plane-parallel light; (b) polarized light.

Inclusions

Sample is rich in inclusions. Most inclusions are primary, single-phase liquid (L). Less than ca. <0.01 % of inclusions contain bubbles. Inclusions are intra- and inter-crystalline.

Mostly, inclusions have elongated shape and are associated with crystal growth surface. In places, such inclusions become very large (Fig. 17); in this case they commonly have irregular shapes. In terms of the volume, such accidental inclusions by far 'overweight' the volume that can be released from many typical small primary inclusions (Fig. 18a).

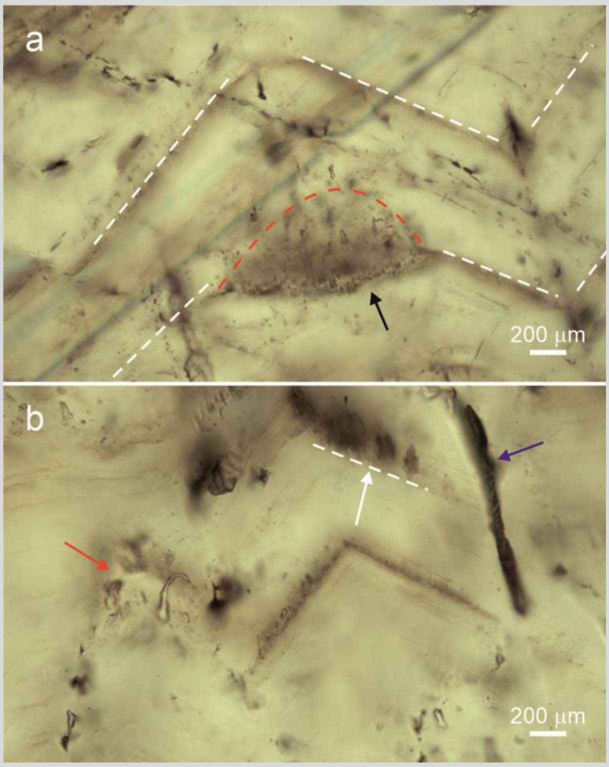

Fig. 18. Section SÜ-3: Primary inclusions. (a) 3D 'cloud' of liquid inclusions formed in front of the advancing crystal edge (red dashed line; black arrow); (b) variously shaped inclusions, formed at the growth surfaces of the two adjacent crystals: in one case large irregularly shaped inclusions have formed (red arrow); in the other – numerous small elongate inclusions were trapped (white arrow); another large tubular inclusion was trapped along the compromise crystal growth boundary (blue arrow). Former crystal growth surface is emphasized by white dashed line.

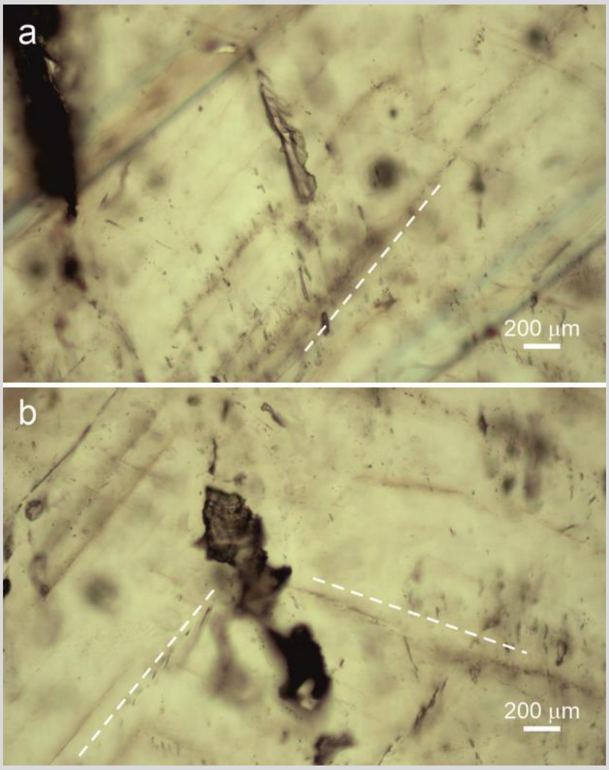

Fig. 17. Section SÜ-3: Large elongated inclusions associated with crystal growth. (a) elongate single-phase liquid (L) inclusion, originated at former crystal growth surface; (b) large irregularly shaped inclusion associated with edge of the crystal (presently vapor-filled because the inclusion was intersected by surface of the section and leaked). Former crystal growth surfaces are emphasized by white dashed lines.

Apparently, formation of large vs. small inclusions in this sample is a stochastic process: in some cases, one can observe drastically different shapes and sizes of inclusions forming at the advancing crystal faces of adjacent crystals (Fig. 18b).

Overall interpretation of SÜ-3

Calcite. The character of calcite (fabrics) suggests its low temperature origin, and is consistnet with subaqueous deposition of calcite.

Fluid inclusions. Most of inclusions in this sample are primary. Both intra- and inter-crystalline inclusions are present. The spectrum

of sizes of inclusions is quite wide; there are sporadic very large water-filled inclusions, commonly primary.

Temperature of formation. Based on the character of fluid inclusions, calcite in this sample formed at relatively low temperature (less than *approximately* 40°C).

Sample SÜ-4

Calcite fabric

Calcite in thus samples has variable fabrics ranging from columnar to mosaic (Fig. 19).

Inclusions

Inclusions are abundant in this sample. Most of them can be classed as primary.

Inclusions are single-phase liquid, aqueous. Similarly to sample SÜ-3, they show a wide spectrum of sizes.

Unlike all other samples, this calcite rarely shows planar crystal faces. Accordingly, in this sample we do not see inclusions, associated with former free crystal growth surfaces. In contrast, inclusions

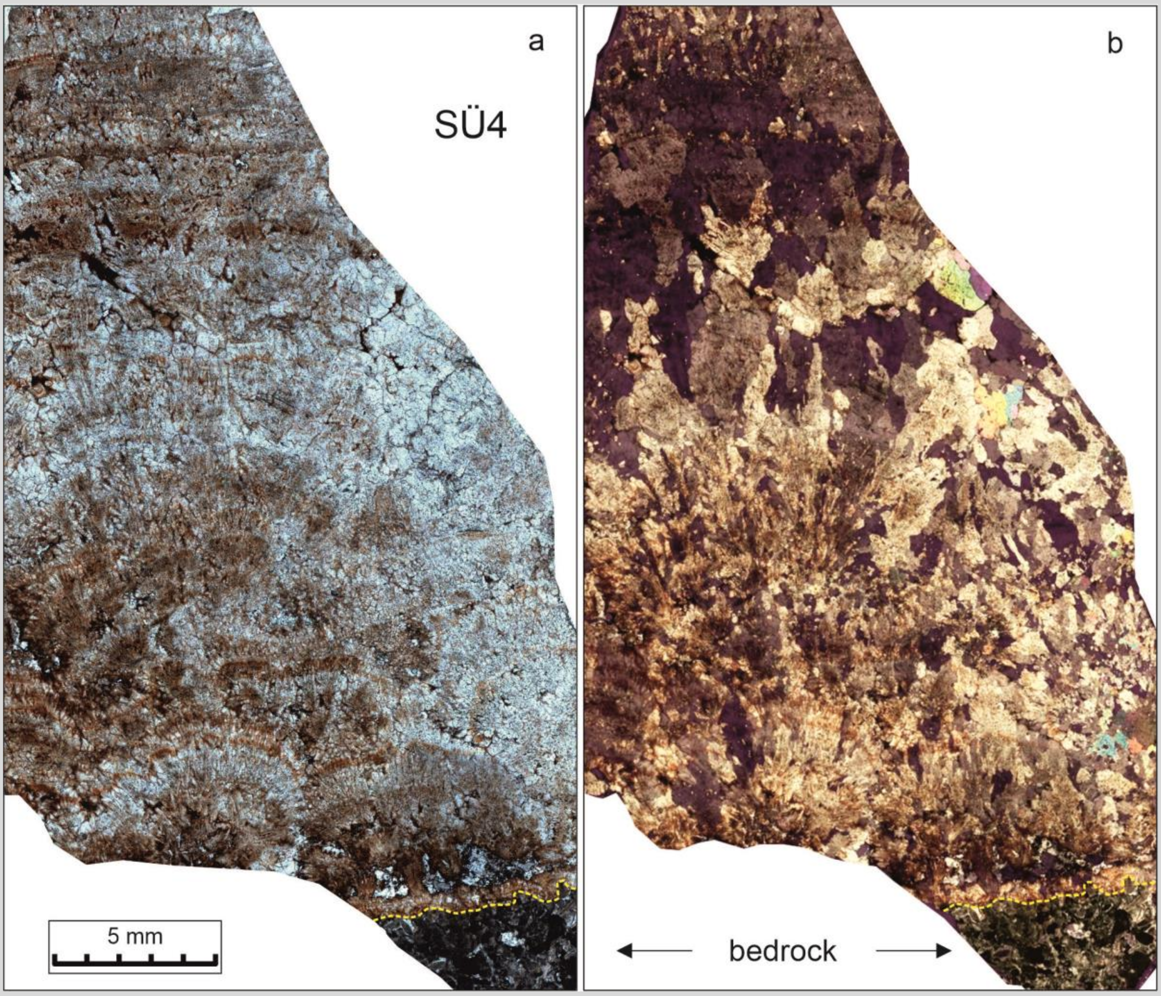

Fig. 19. Section SÜ-4: (a) plane-parallel light; (b) polarized light.

commonly form 3D ‘clouds’ in core parts of crystals (Fig. 20). Compromise boundaries between crystals also contain inclusions.

Most of the inclusions are single-phase liquid, aqueous (Fig. 21a). Two-phase inclusions are not common in this sample. I have found only one group of two-phase (LV) inclusions occur, showing somewhat consistent V/L ratios (Fig. 21b).

Overall interpretation of SÜ-4

Calcite. The character of calcite (fabrics) is consistent with a low temperature origin.

Fluid inclusions. The abundance of fluid inclusions suggests relatively fast growth and formation of many crystals with abundant growth defects, similarly to travertines. The absolute majority of inclusions is single-phase, aqueous. The group of two-phase inclusions shown in Fig. 21b, is, most likely, an artefact, related to local volume change (stretching) of inclusions. The latter can have natural (tectonic stress) or artificial causes (sample preparations).

Temperature of formation. The host calcite was formed ate relatively low temperature (less than *approximately* 40°C).

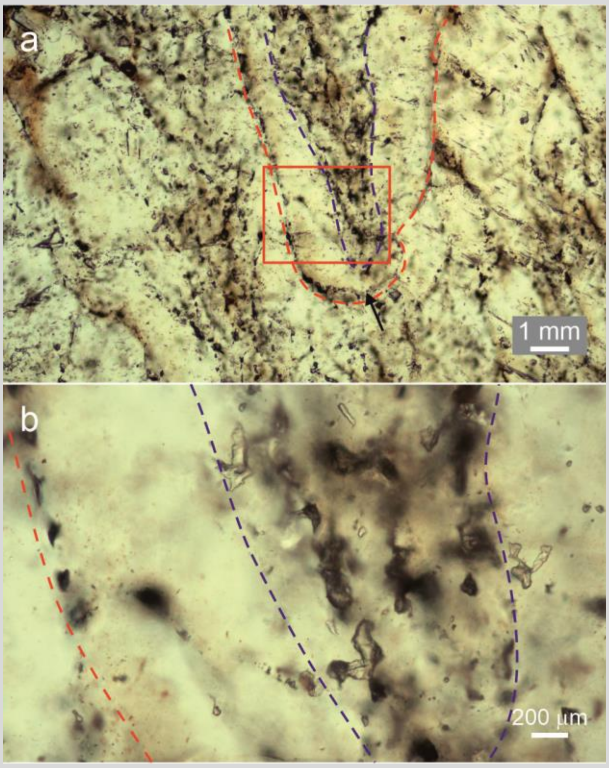

Fig. 20. Section SÜ-4: (a) 3D ‘cloud’ of inclusions in central part of the crystal (delineated by blue dashed line); the compromise growth boundary of the crystal (red dashed line) also contains fluid inclusions; (b) close-up on the red rectangle shown in **a**.

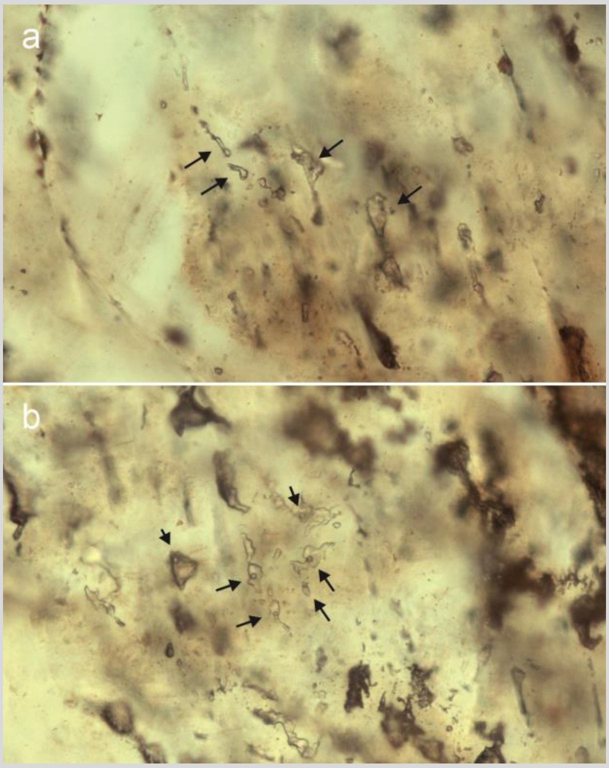

Fig. 21. Section SÜ-4: (a) single-phase liquid, aqueous inclusions (arrows) in the core part of the crystal; (b) Group of two-phase (LV) inclusions (arrows) showing somewhat consistent V/L ratios).

Sample VK-1

Calcite

Calcite aggregate in section VK-1 is built up of nearly isometric euhedral to subhedral crystals, densely packed into a mosaic fabric (Fig. 22). Sizes of individual crystals range between ca. 1 and 10 mm.

Growth zones are pronounced in most of the crystals (Fig. 22a); they do not transcend crystal boundaries.

Under partly crossed Nicols, crystals reveal lamellar twinning (Fig. 22b), indicating that the aggregate has experienced mechanical stress.

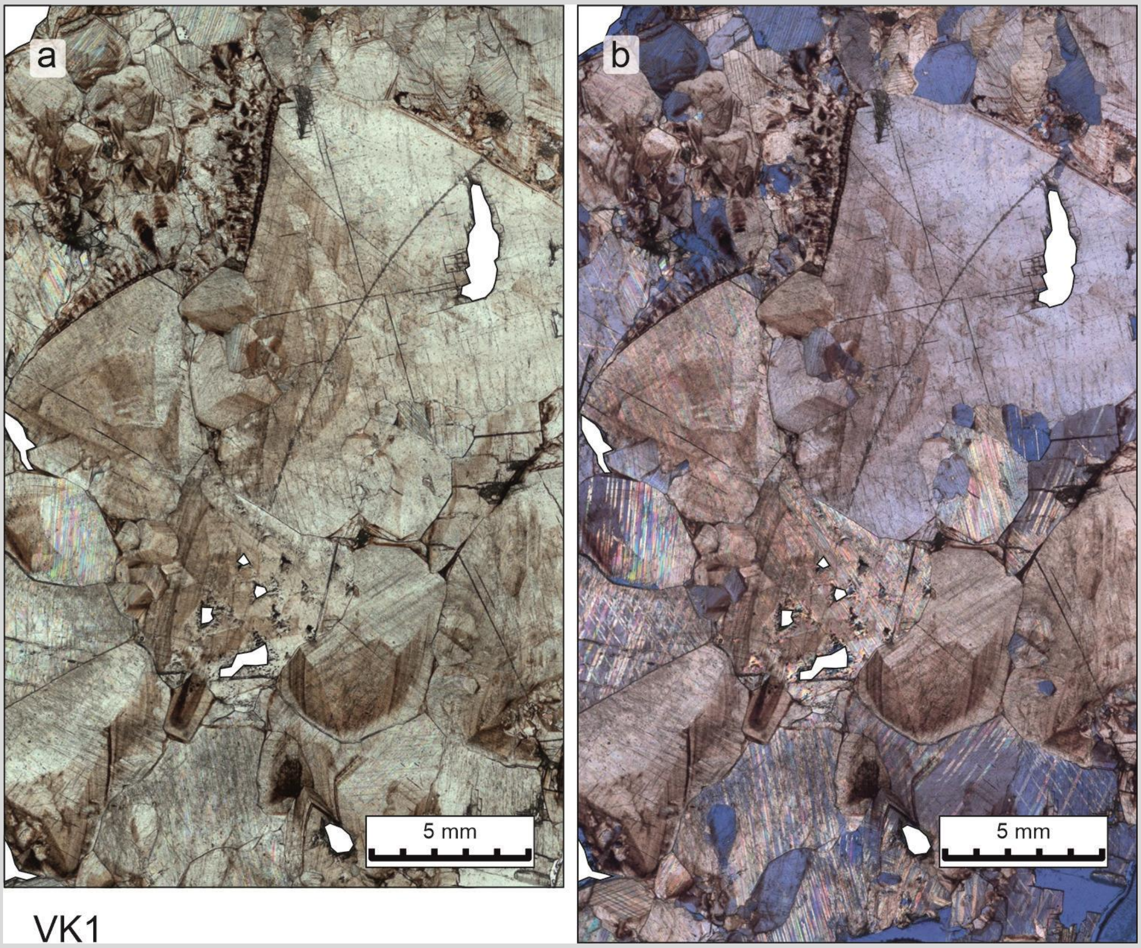

Fig. 22. Section VK-1: (a) plane-parallel light; (b) polarized light.

Filaments
Not too abundant, but persistent filaments are noted in the crystals. Commonly, they are oriented along the former crystal faces (Fig. 23), confirming entrapment during crystal growth.

Inclusions
Calcite hosts relatively large (10–30 μm in size) primary inclusions. All inclusions are single-phase aqueous. Their primary character is revealed by their characteristic waisted shape (directional inclusions) (Fig. 24a). There are also FIAs of inclusions, associated with slightly undulous fractures, roughly aligned with the direction of crystal growth. Origin of these inclusions is unclear (primary or secondary). Inclusions in such FIAs are small (1–5 μm) elongate (Fig. 24a).

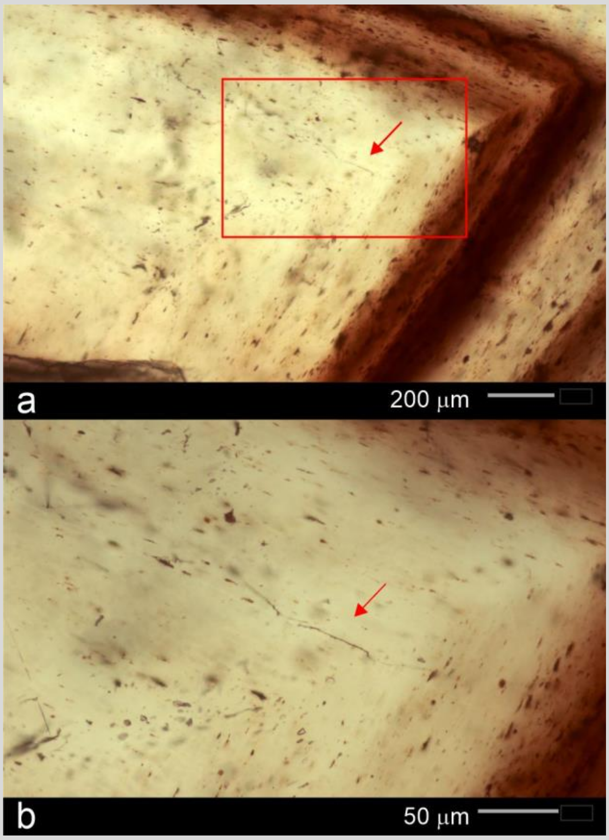

Fig. 23. a – Growth zoning and filaments trapped on the surface of growing calcite crystal; b – close up on filament.

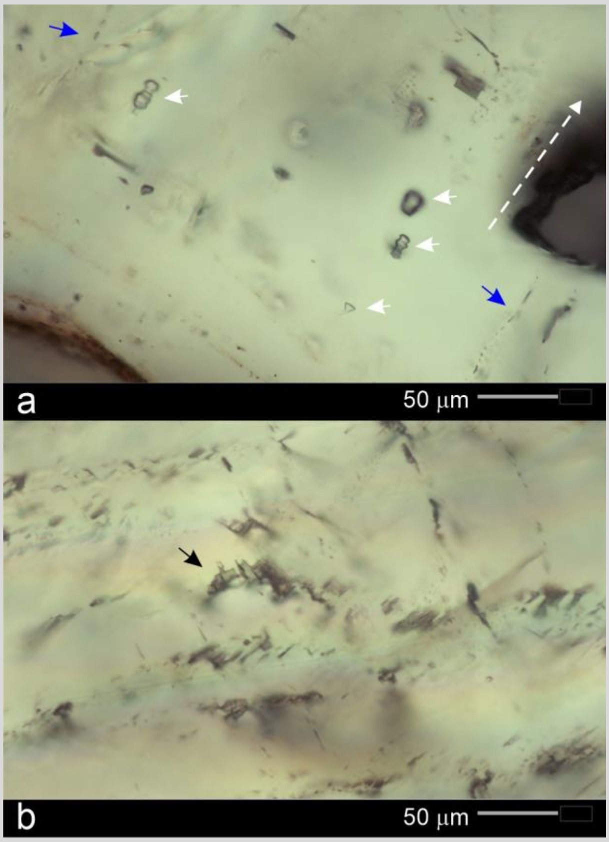

Fig. 24. a – Relationships between primary (white arrows) and secondary (blue arrows) fluid inclusions. Note characteristic shapes of primary fluid inclusions. Dashed white arrows indicate the direction of crystal growth (face advancement). b – Inclusions with highly irregular shape; character indeterminate (black arrow). All inclusions are single-phase, aqueous.

In places, FIAs comprising inclusions with highly irregular shapes are present (Fig. 24b). They may have a relatively large volume (commensurate with the volume of primary waisted inclusions). Since these FIAs do not exhibit clear relationships with crystal growth, origin of these inclusions is indeterminate.

Overall interpretation of VK-1
Calcite. The fabrics of this sample suggests deposition in subaqueous conditions.

Fluid inclusions. Most of the water contained in this sample resides in intra-crystalline primary inclusions. Such inclusions are single-phase,

aqueous. Secondary inclusions are present, but volumetrically insignificant. There are sporadic occurrences of inclusions of indeterminate origin.

Traces of biological activity. Filaments, representing remnants of contemporaneous life (likely, fungi) were observed entrapped in calcite. This suggests relatively low-temperature environment.

Temperature of formation. Based on exclusively single-phase character of primary inclusions, the host calcite was formed at relatively low temperatures (less than *approximately* 40°C).

Sample VK-2

Calcite

Calcite aggregate in section VK-2 has compact columnar fabric (Fig. 25). It also exhibits relatively smooth growth zones, transcending boundaries of competitively growing columnar crystals.

The aggregate is subject to intense twinning (Fig. 25), indicating that it has experienced mechanical stress.

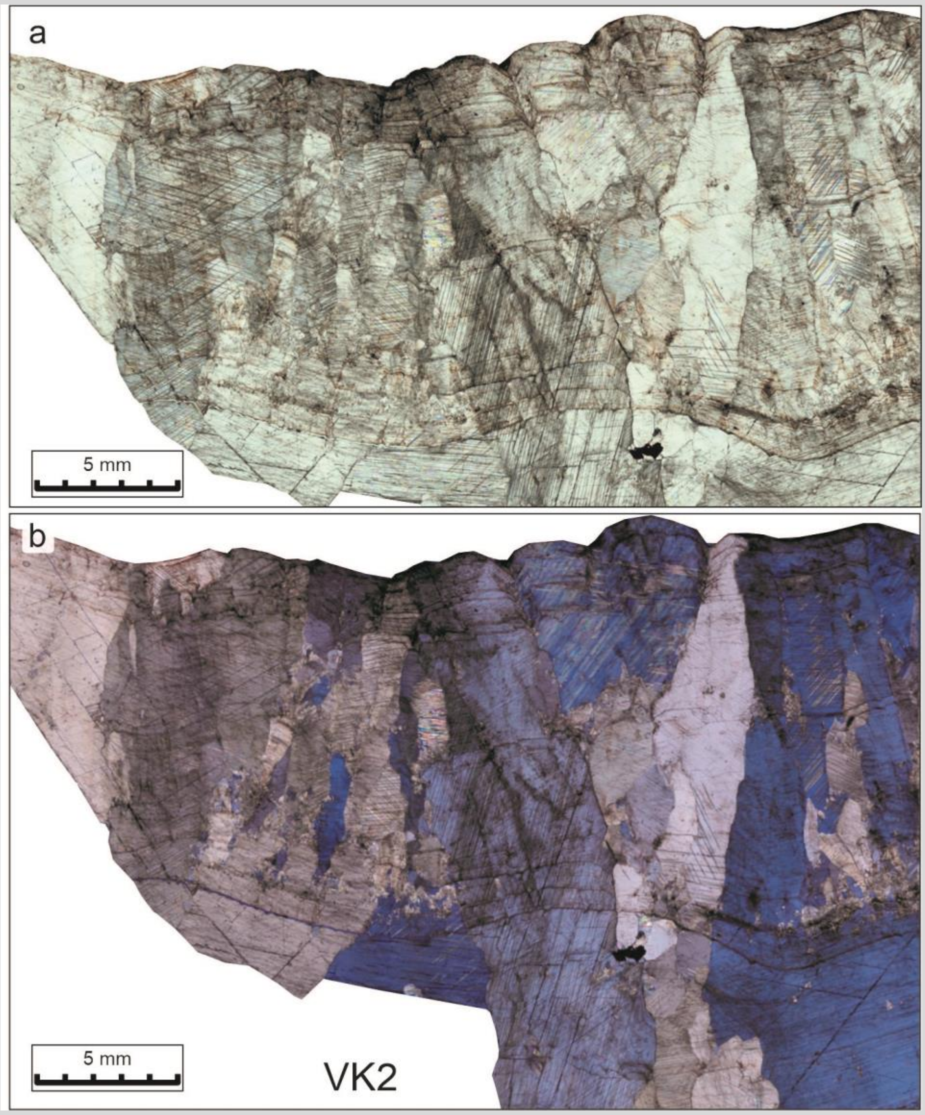

Fig. 25. Section VK-2: (a) plane-parallel light; (b) polarized light.

Filaments

Persistent, but not abundant filaments are present in the host calcite (Fig. 26).

Inclusions

Most of the observed fluid inclusions in this sample are primary. Part of them have characteristic shapes (directional inclusions; Fig. 27a); they are persistent but not abundant. Another type is represented by relatively small, very irregularly shaped inclusions, which form dense 3D “clouds” in palisade crystals (Fig. 27b).

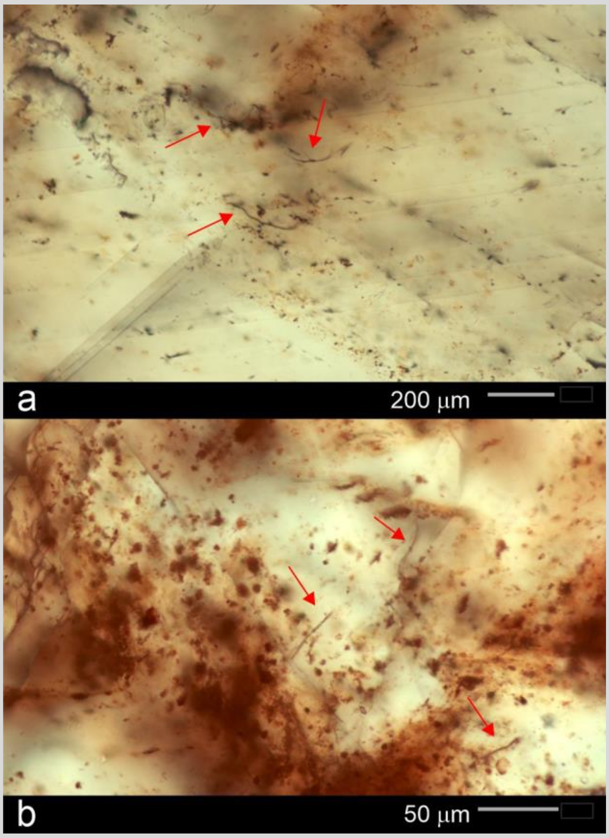

Fig. 26. Filaments (red arrows) trapped on the surface of growing calcite crystal.

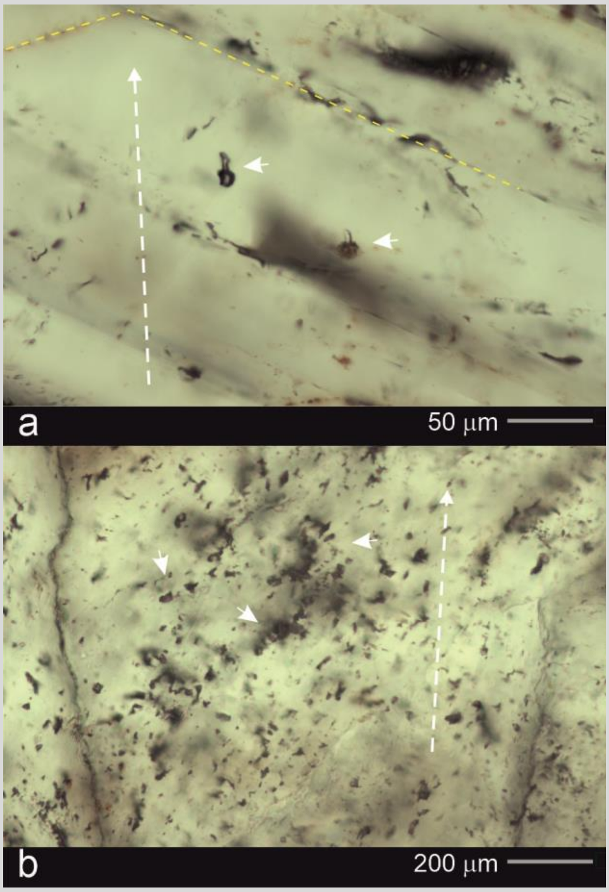

Fig. 27. Primary fluid inclusions (white arrows): a – characteristically shaped directional inclusions; b – inclusions with highly irregular shapes forming 3D “clouds” in palisade crystals. Direction of calcite growth is indicated by dashed white arrow. Former calcite growth surface is marked by yellow dashed line in a.

Most of the inclusions observed in this section are intra-crystalline. Adjacent calcite subcrystals are perfectly coalesced, so that virtually no fluid inclusions are trapped along the compromise growth boundaries.

Overall interpretation of VK-2

Calcite. The fabrics of this sample does not provide a clear indication of conditions of formation; it is consistent with both water film-flow (speleothemic) and submerged origins. Based on large sizes of columnar crystals, subaqueous deposition is more likely.

Fluid inclusions. Most of the water contained in this sample resides in intra-crystalline primary inclusions. Inclusions are single-phase, aqueous.

Traces of biological activity. Filaments, representing remnants of contemporaneous life

(likely, fungi) were observed entrapped in calcite. This suggests relatively low-temperature environment.

Temperature of formation. Based on exclusively single-phase character of primary inclusions, the host calcite was formed ate relatively low temperature (less than *approximately* 40°C).

Sample VK-6

Calcite

Calcite aggregate in section VK-6 has a complex build-up featuring, in the lower part of the section, euhedral crystals, their columnar overgrowth, and deposition of opaque sediments in the interstices (Fig. 28). In the upper part of the section calcite morphs into an aggregate of crystals showing compact columnar fabric (Fig. 28a) and pronounced growth zones. The latter range from smooth to zig-zagged, defining the former calcite crystal faces. This sequence of fabrics is intersected by a late-generation branching calcite veinlet (Fig. 28).

Columnar calcite in the upper part of the sequence shows rather intense mechanical twinning (Fig. 29a).

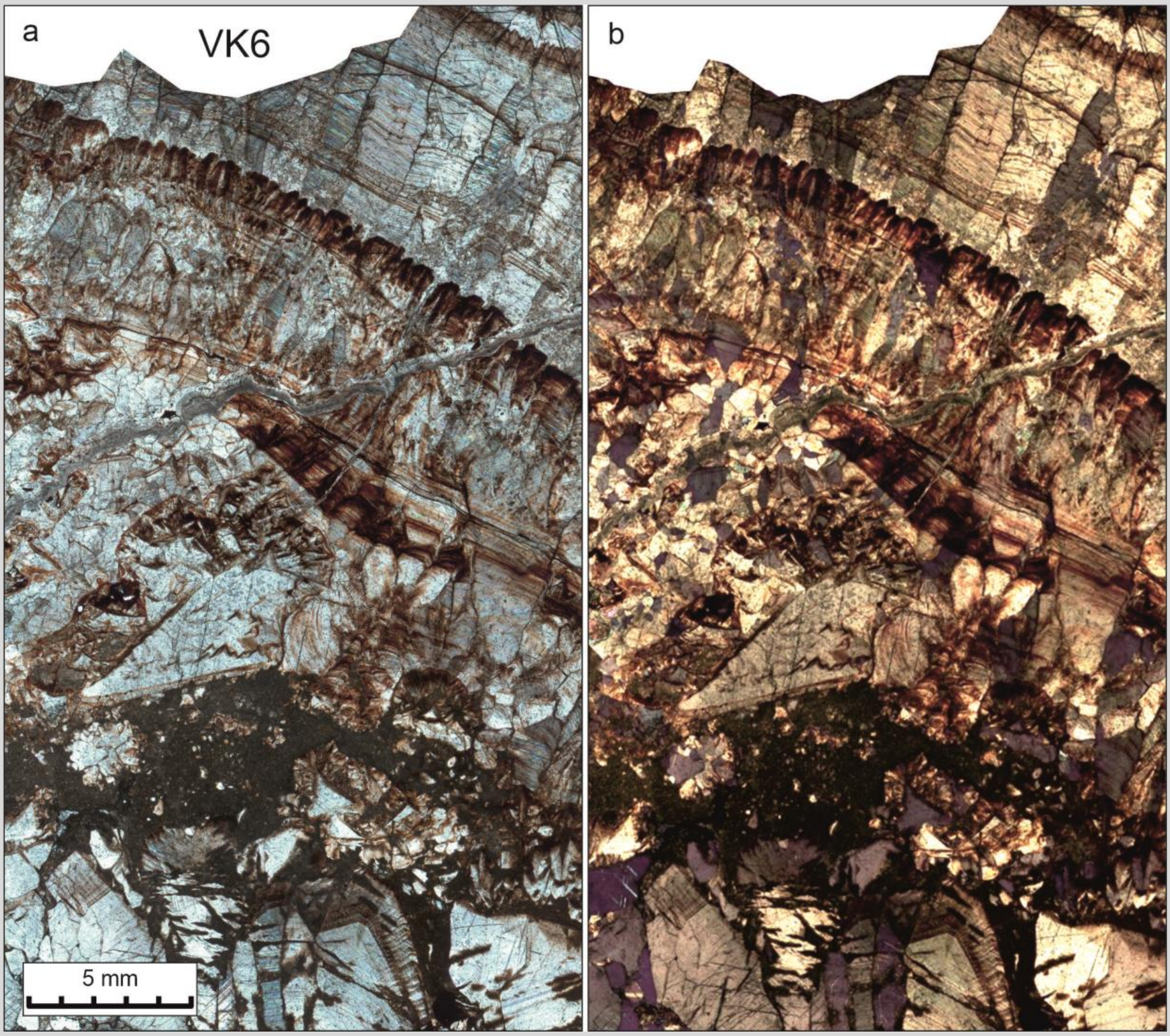

Fig. 28. Section VK-6: (a) plane-parallel light; (b) polarized light.

Filaments

Not too abundant, but persistent filaments are noted in the crystals. Commonly, they are oriented along the former crystal faces (Fig. 30), confirming entrapment during crystal growth.

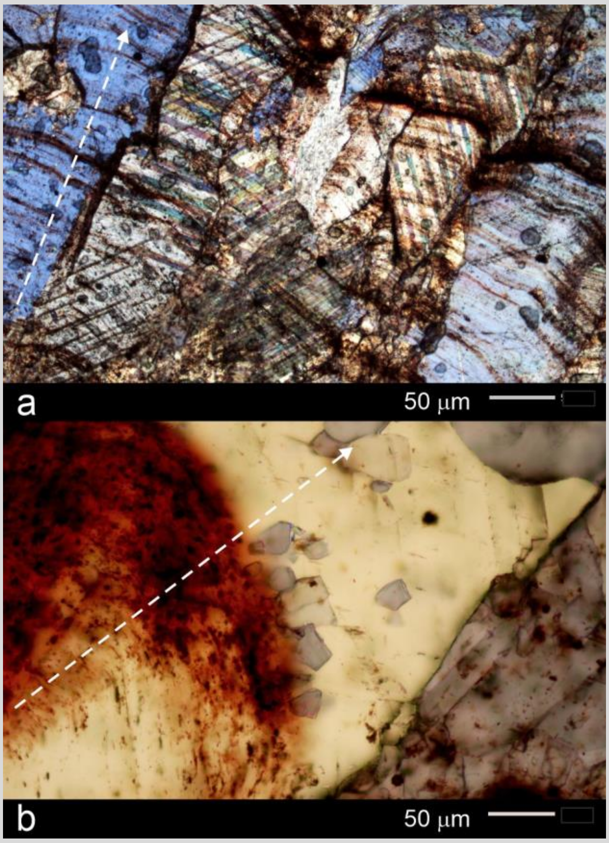

Fig. 29. a – Compact columnar fabric in the upper part of the section. Note intense mechanical twinning (polarized light). b – Nucleation of small subhedral, nearly isometric calcite crystals during the growth of the "main" calcite crystal in the lower part of the section. Dashed white arrows indicate the direction of crystal growth (face advancement).

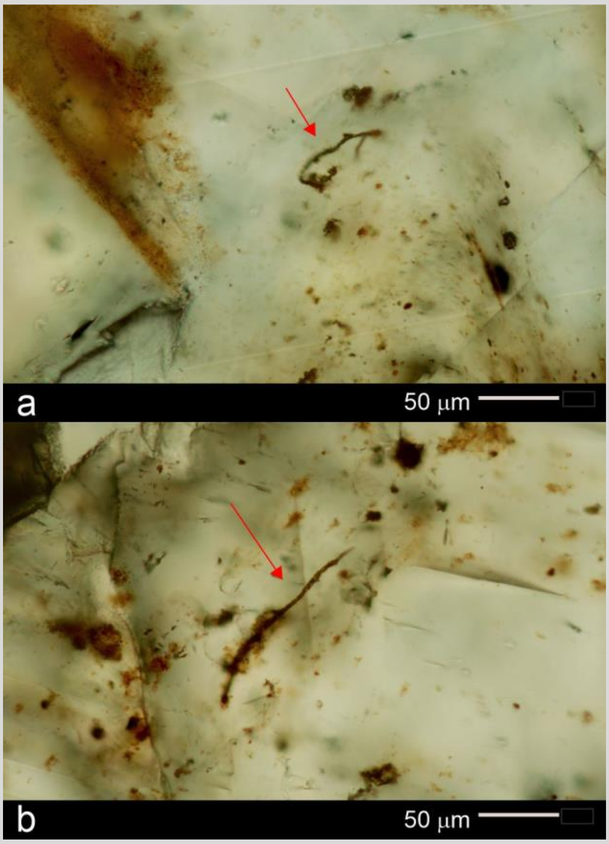

Fig. 30. Filaments in calcite (red arrows).

Inclusions

Calcite hosts relatively large (10–30 μm in size) primary inclusions. All primary inclusions are single-phase aqueous. Their primary character is revealed by their characteristic waisted shape (directional inclusions; Fig. 31b and 32a). There are also FIAs associated with slightly undulous fractures, extending all the way to the crystal surface (Fig. 31a, b).

Because these FIAs are roughly parallel to the direction of crystal growth, it is difficult to ascertain their origin: they can be secondary, or they can be primary intra-crystalline inclusions (trapped between adjacent subcrystals). These indeterminate inclusions are small (2–5 μm) elongate (Fig. 31b).

In this sample a FIA comprising two-phase inclusions with apparently consistent and small V/L ratios of <<0.1 (Fig. 32b) was observed. Bubbles are present in vacuoles that have flattened shape (inclusions with this shape are particularly prone to stretching upon heating). This suggest that the calcite of sample VK-6 could have been exposed, *after its formation*, to moderately high temperatures. This is also consistent with the presence of cross-cutting veinlet (see Fig. 28). Inclusions, shown in Fig. 32b most likely represent the result of re-

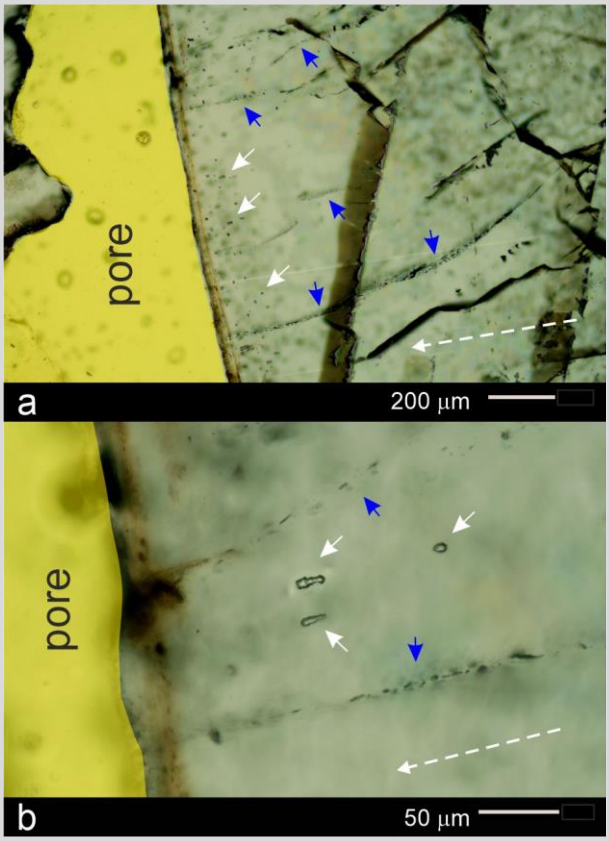

Fig. 31. Relationships between primary (white arrows) and indeterminate (secondary or primary inter-crystalline; blue arrows) fluid inclusions. Note characteristic shapes of primary fluid inclusions. Dashed white arrows indicate the direction of crystal growth (face advancement).

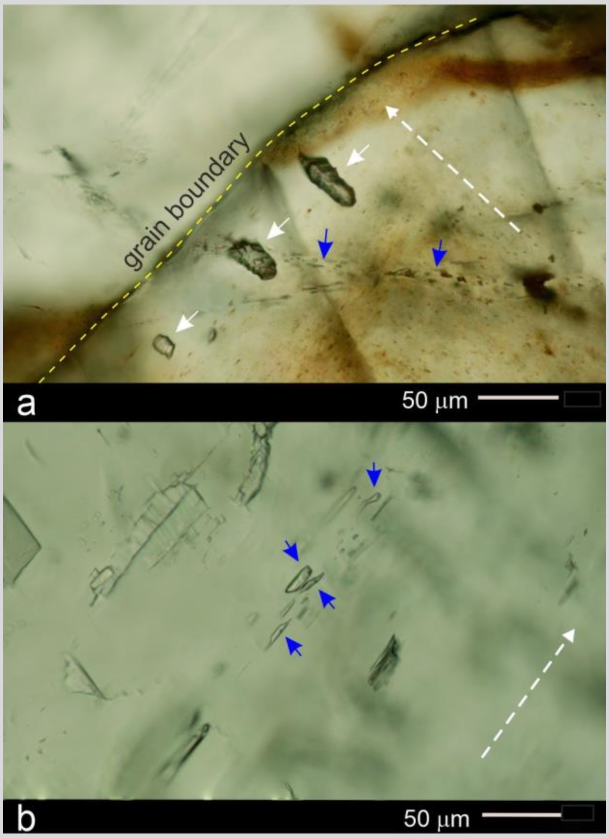

Fig. 32. Different character of primary (white arrows) and secondary (blue arrows) inclusions: a – large primary single-phase aqueous inclusions elongate in the direction of crystal growth and a trail of small, likely secondary inclusions; b – FIA of secondary (?) two-phase inclusions with seemingly consistent V-L ratios. Dashed white arrows indicate the direction of crystal growth (face advancement).

equilibration of initially single-phase aqueous inclusions caused by this thermal exposure.

Overall interpretation of VK-6

Calcite. The fabrics of this sample suggests deposition in subaqueous conditions.

Fluid inclusions. Fig. 33 provides a good summary of fluid inclusion types in sample VK-6. Primary inclusions are mostly intra-crystalline, single-phase, aqueous. Such inclusions volumetrically dominate. Secondary inclusions are present, but volumetrically insignificant.

Traces of biological activity. Filaments, representing remnants of contemporaneous life (likely, fungi) were observed entrapped in calcite. This is consistent with relatively low-temperature environment.

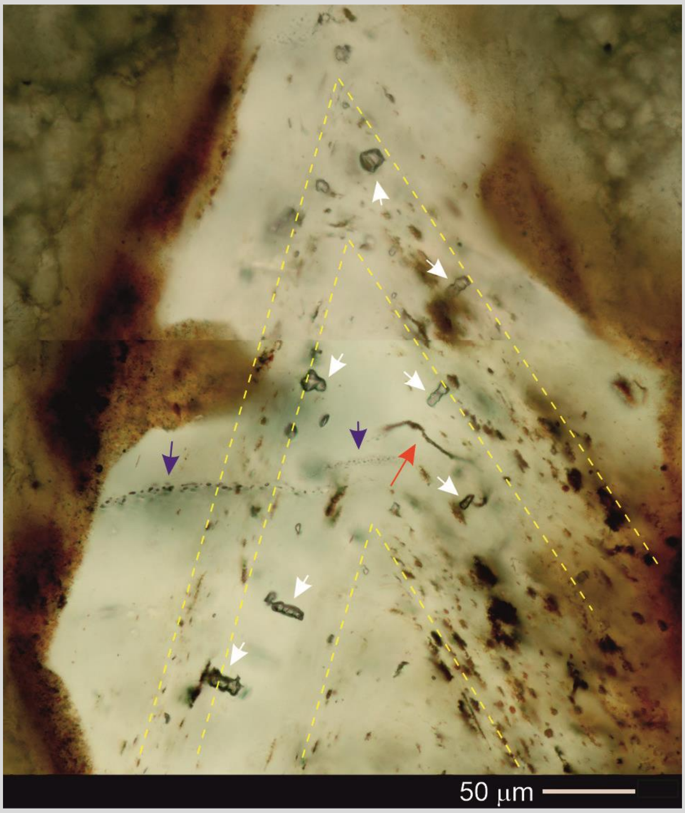

Fig. 33. Inclusions and filaments in individual crystal. Primary inclusions (white arrows) aligned along growth zones (emphasized by yellow dashed lines); secondary inclusions (blue arrows) along curvilinear trails; filament (red arrow)

Temperature of formation. Based on exclusively single-phase character of primary inclusions, the host calcite was formed ate relatively low temperature. Yet, the presence of FIAs of presumably secondary inclusions that contain shrinkage bubbles suggest post-depositional exposure to elevated temperatures. This exposure has led to re-equilibration of part of inclusions, which had flattened shapes and were, therefore, most prone to volume change. Based on the observed V/L ratios in such inclusions, the temperature of thermal fluids was not too high (above *approximately* 40 °C, but not exceeding 70 °C).

References

Frisia S., Borsato A., Fairchild I.J., McDermott F. (2000): Calcite fabrics, growth mechanisms, and environments of formation in speleothems from the Italian Alps and Southwestern Ireland. *Journal of Sedimentary Research*, 70(5): 1183-1196, DOI: 10.1306/022900701183

Goldstein R.H., Reynolds T.J. (1994): Systematics of fluid inclusions in diagenetic minerals. SEPM Short Course 31, Tulsa, Oklahoma

Cathodoluminescence microscopic images (scale bars: 1 mm)

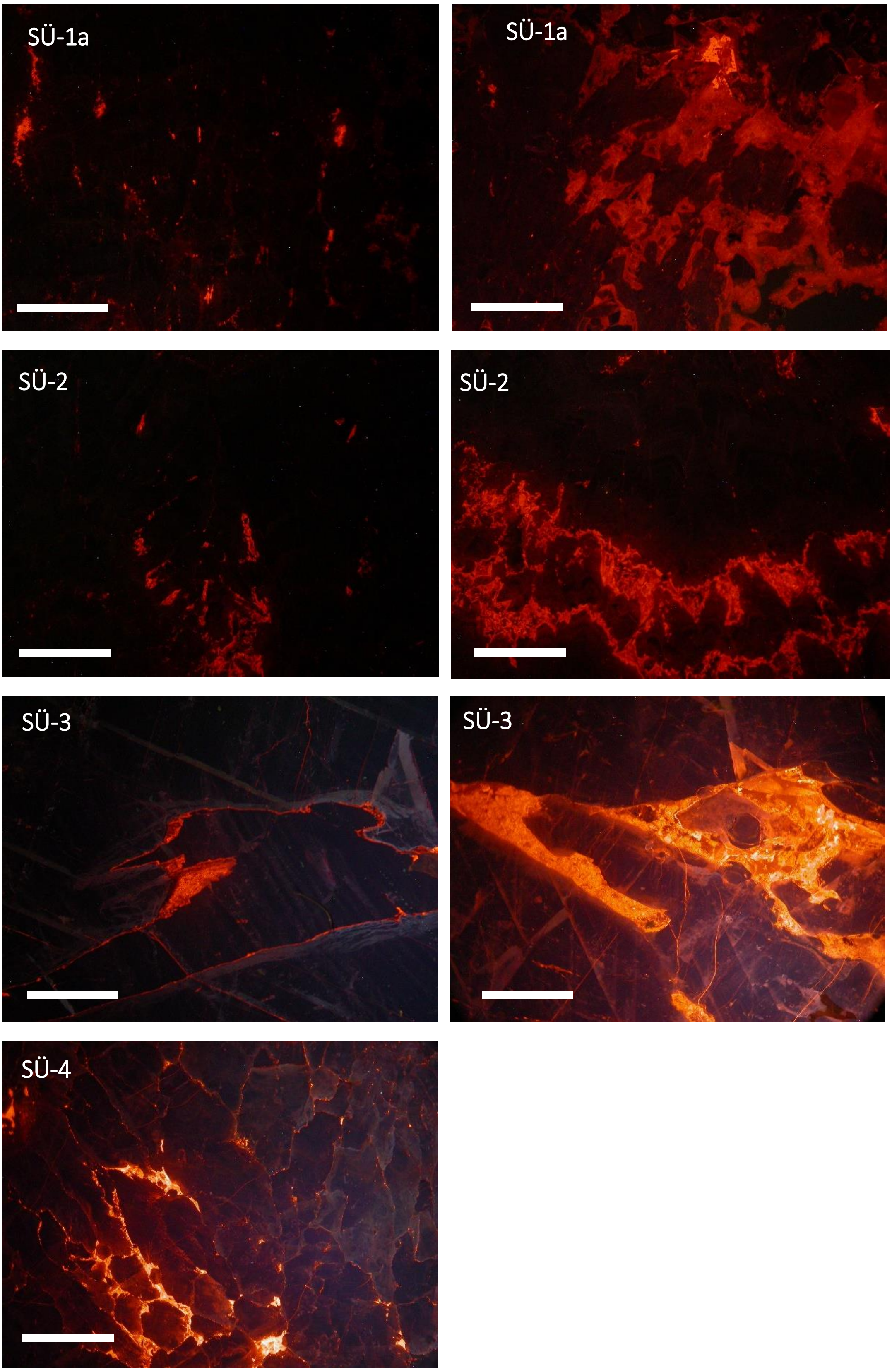

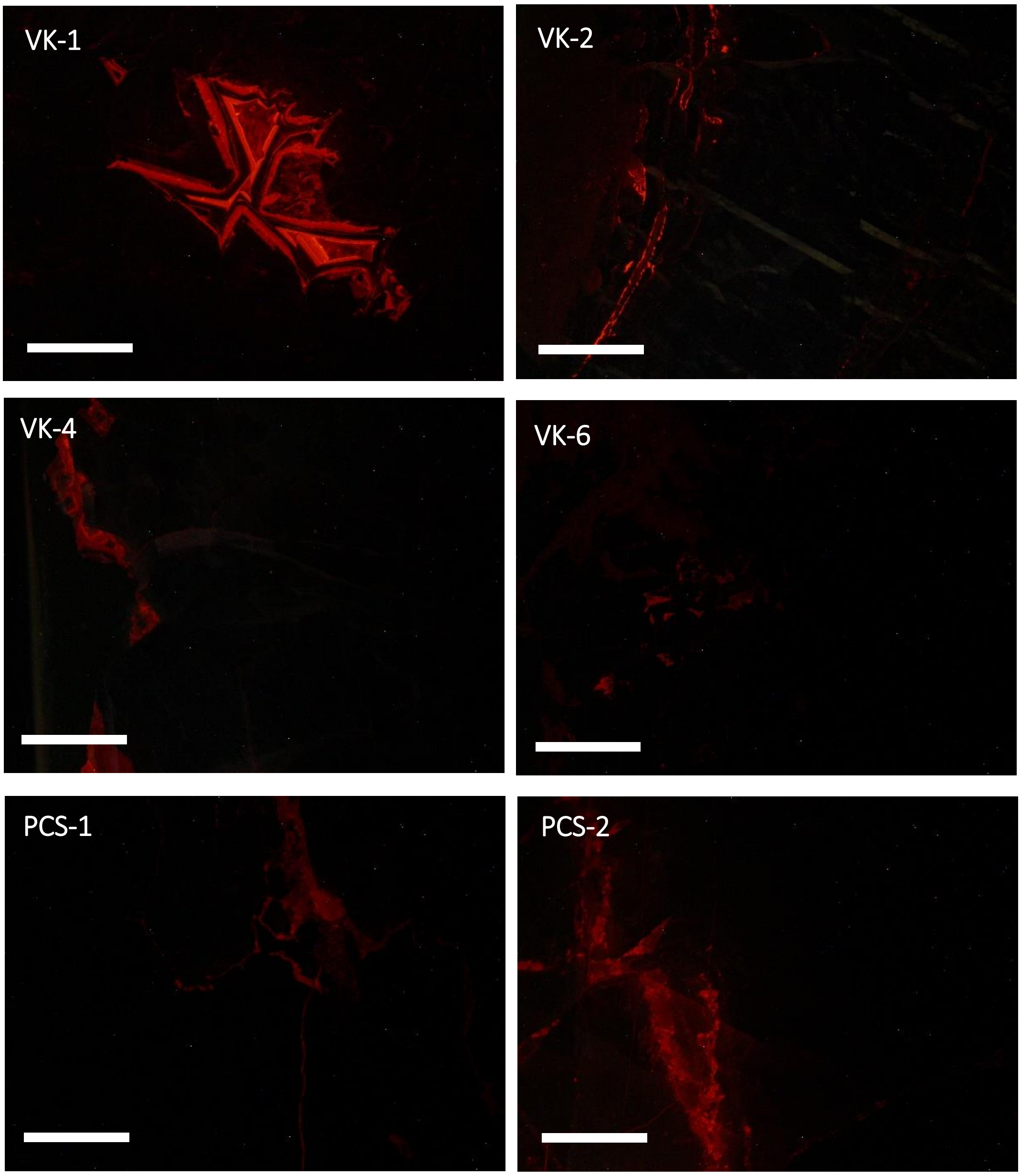
VK-1
VK-2
VK-4
VK-6
PCS-1
PCS-2
